# IκBα mediates prostate cancer cell death induced by combinatorial targeting of the androgen receptor

**DOI:** 10.1186/s12885-016-2188-2

**Published:** 2016-02-23

**Authors:** Sarah Louise Carter, Margaret Mary Centenera, Wayne Desmond Tilley, Luke Ashton Selth, Lisa Maree Butler

**Affiliations:** Dame Roma Mitchell Cancer Research Laboratories, Adelaide Prostate Cancer Research Centre and Freemason’s Foundation Centre for Men’s Health, School of Medicine, University of Adelaide and Hanson Institute, Adelaide, SA 5005 Australia; Cancer Theme, South Australian Health and Medical Research Institute, Adelaide, SA 5001 Australia

**Keywords:** Prostate cancer, Androgen receptor, Combination therapy, IκBα

## Abstract

**Background:**

Combining different clinical agents to target multiple pathways in prostate cancer cells, including androgen receptor (AR) signaling, is potentially an effective strategy to improve outcomes for men with metastatic disease. We have previously demonstrated that sub-effective concentrations of an AR antagonist, bicalutamide, and the histone deacetylase inhibitor, vorinostat, act synergistically when combined to cause death of AR-dependent prostate cancer cells.

**Methods:**

In this study, expression profiling of human prostate cancer cells treated with bicalutamide or vorinostat, alone or in combination, was employed to determine the molecular mechanisms underlying this synergistic action. Cell viability assays and quantitative real time PCR were used to validate identified candidate genes.

**Results:**

A substantial proportion of the genes modulated by the combination of bicalutamide and vorinostat were androgen regulated. Independent pathway analysis identified further pathways and genes, most notably *NFKBIA* (encoding IκBα, an inhibitor of NF-κB and p53 signaling), as targets of this combinatorial treatment. Depletion of IκBα by siRNA knockdown enhanced apoptosis of prostate cancer cells, while ectopic overexpression of IκBα markedly suppressed cell death induced by the combination of bicalutamide and vorinostat.

**Conclusion:**

These findings implicate IκBα as a key mediator of the apoptotic action of this combinatorial AR targeting strategy and a promising new therapeutic target for prostate cancer.

**Electronic supplementary material:**

The online version of this article (doi:10.1186/s12885-016-2188-2) contains supplementary material, which is available to authorized users.

## Background

Prostate cancer is the most commonly diagnosed cancer, and the second leading cause of cancer-related death, in men in the developed world [1]. Since Huggins, Stevens and Hodges [2] demonstrated that prostate epithelial cells require androgens for growth and survival, the mainstay of treatment for men with metastatic prostate cancer has been suppression of testosterone production by surgical or medical castration, a strategy termed androgen deprivation therapy (ADT). Whilst these treatment modalities are initially effective (reviewed in [3]), most patients eventually relapse with castrate-resistant prostate cancer (CRPC), which is incurable and the primary cause of mortality associated with this disease.

It is now well established that the mediator of androgen action, the androgen receptor (AR), plays a key role in the progression of prostate cancer following ADT, despite castrate levels of circulating testosterone. A number of mechanisms, including increased levels of the AR mRNA or protein [4–8], mutation of the *AR* gene to produce more active or promiscuous forms of the receptor [9–14], altered levels of AR coregulators (reviewed in [15]), the expression of constitutively active AR splice variants [16–18], and adrenal and intratumoral biosynthesis of androgens [19–23], explain continued AR signaling during ADT. As many of these mechanisms are refractory to conventional ADT, there is considerable impetus to develop new and more potent agents targeting the androgen signaling axis. Two such agents are enzalutamide (MDV-3100), a novel AR antagonist that has demonstrated clinical activity in men who have failed both ADT and docetaxel-based chemotherapy [24], and abiraterone acetate, which targets an enzyme required for adrenal and intratumoral androgen biosynthesis. Phase III clinical trials demonstrated that these agents extend median survival of men with advanced CRPC by several months and both have received FDA approval [25].

Despite the success of enzalutamide and abiraterone, it is accepted that treatment with these agents remains essentially palliative, and that combinatorial treatment strategies targeting multiple cellular pathways in addition to androgen signaling are more likely to improve outcomes for men with CRPC. One such combination therapy comprises the AR antagonist bicalutamide and the histone deacetylase (HDAC) inhibitor vorinostat, which act synergistically together to cause death of cell line models of prostate cancer [26]. Vorinostat has a global effect on the acetylation of histones and other proteins within the cell but also reduces AR levels and activity and thereby directly targets androgen signaling [26]. The aim of this study was to interrogate the molecular mechanisms underlying the synergistic action of bicalutamide and vorinostat in prostate cancer. Through expression profiling and functional studies, we identified *NFKBIA* (IκBα) as a critical mediator of this therapy, and in doing so provided novel insight into AR signaling and how this might be effectively targeted in prostate cancer.

## Methods

### Cells and reagents

LNCaP human prostate cancer cells were purchased from the American Type Culture Collection (ATCC, Rockville, MD, USA), maintained in RPMI 1640 supplemented with 10 % fetal bovine serum (FBS) and used within a range of 20–40 passages. VCaP human prostate cancer cells were purchased from the ATCC, maintained in DMEM supplemented with sodium pyruvate, non-essential amino acids and 10 % FBS, and used within 60–70 passages. Vorinostat was obtained from Merck (New Jersey, USA) and dissolved in DMSO. Bicalutamide was obtained from Astra Zeneca (London, UK) and dissolved in ethanol. Cycloheximide was obtained from Sigma (St. Louis, MO, USA) and dissolved in DMSO. Anti-AR (N-20), anti-prostate specific antigen (PSA; C-19) and anti-heat shock protein 90 (HSP90; H-114) antibodies were obtained from Santa Cruz Biotechnology (Santa Cruz, CA, USA). Anti-IκBα antibody was obtained from Cell Signaling Technology Inc (Danvers, MA, USA). Anti-αtubulin antibody was obtained from Merck Millipore (Billerica, MA, USA). Horseradish peroxidase conjugated anti-rabbit, anti-mouse, and anti-sheep/goat secondary antibodies were obtained from DAKO (Botany, NSW, Australia). Non-specific, scrambled siRNA and ON-TARGETplus siRNAs targeting *NFKBIA* were purchased from Dharmacon (Lafayette, CO, USA) and the *NFKBIA-IRES-eGFP* lentiviral ORF plasmid was purchased from GeneCopoeia (Rockville, MD, USA). The pLV410 eGFP lentiviral ORF plasmid was kindly provided by Dr. Philip Gregory (University of Adelaide, Adelaide, Australia).

### Cell viability assays

LNCaP or VCaP cells were seeded in triplicate in 24-well plates, and allowed to attach overnight before the growth medium was replaced with medium containing vehicle control or the indicated concentrations of vorinostat, bicalutamide, or the two agents in combination. Doses were calculated based on individual dose-response curves for each agent and cell line to ensure consistency in the antiproliferative response between different cell lines ([26], Additional file 1: Figure S1). Cells were counted every 2 days using a hemocytometer and cell viability was assessed using Trypan blue dye exclusion. For sequential treatments, cells were treated with drug one for 24 h, at which point the treatment medium was removed and replaced with drug two for 48 h. To assess the effect of cycloheximide, cells were pre-treated with 10 μM cycloheximide for 1 h, which was then removed and replaced with treatment medium. Wash out experiments were performed by allowing the treatment medium to remain on the cells for 1, 2, 4, 6, 8, 16, or 24 h, at which point it was removed and replaced with drug-free medium. At each end-point cells were counted using a hemocytometer and viability assessed as above.

### Microarray analysis

LNCaP cells were cultured with vehicle control, 1 μM vorinostat, 5 μM bicalutamide, or the two agents in combination for 6 h. Adjustment of the dose of bicalutamide compared to the initial cell viability assays was necessary to ensure consistency in terms of cell death between the two experiments, due to variation in the sensitivity of LNCaP cells to this agent over time. Total RNA was extracted from the cells using Trizol reagent (Life Technologies, Carlsbad, CA, USA), and RNA integrity was analyzed on an Agilent Systems Bioanalyzer. RNA from cells treated with the combination of vorinostat and bicalutamide was compared with RNA from cells treated with either vehicle control or either of the agents individually using Affymetrix Human GeneChip ST 1.0 arrays at the Adelaide Microarray Centre, as described previously [27]. Differential gene expression was assessed by ANOVA with the p-value adjusted using a step-up multiple test correction to control the false discovery rate (FDR) [28]. Adjusted *p*-values < 0.05 were considered to be significant.

### Quantitative real-time PCR

Independent RNA samples used to validate the microarray data were generated by culturing LNCaP cells with vehicle control, 1 μM vorinostat, 2.5 μM bicalutamide or the two agents in combination for 3, 6, 9 and 12 h. Total RNA (1 μg) was DNAse treated with Turbo DNA Free (Ambion, Austin, TX, USA), and then reverse transcribed using an iScript cDNA Synthesis Kit (Bio-Rad, Hercules, CA, USA). qRT-PCR was performed with a 1:10 dilution of the cDNA using SYBR green (Bio-Rad) on a CFX Real-Time System (Bio-Rad). geNORM analysis was used to determine appropriate housekeeper genes for each sample set. Microarray RNA was normalized to HPRT1 and RPL19, and the independent sample set was normalized to GUSB and HPRT1. Primer sequences are listed in Additional file 2: Table S1.

### Immunoblotting

LNCaP cells cultured with 2.5 μM bicalutamide or 1 μM vorinostat, individually and in combination, were lysed in radioimmunoprecipitation assay lysis buffer (10 mM Tris–HCl, 125 mM NaCl, 1 mM EDTA, 1 % Triton X-100) supplemented with protease inhibitor cocktail (Roche, Mannheim, Germany). Lysates (20 μg) were electrophoresed through 7.5–15 % SDS-polyacrylamide gels and transferred onto nitrocellulose membranes (GE Healthcare, Buckinghamshire, UK). Membranes were blocked overnight (4 °C) in 3 % non-fat milk powder in Tris-buffered saline containing 0.05 % Tween-20 (TBST). Immunodetection was performed overnight at 4 °C in 3 % non-fat milk powder in TBST using an anti-AR (1:1000) rabbit polyclonal antibody, anti-IκBα (1:1000) mouse monoclonal antibody, or anti-PSA (1:500) goat polyclonal antibody. Antibodies against HSP90 (1:1000, rabbit polyclonal) and α-tubulin (1:1000, mouse monoclonal) were used to assess loading. Proteins were detected with horseradish peroxidase-conjugated secondary antibodies and visualized on autoradiography film using enhanced chemiluminescence detection (GE Healthcare).

### Pathway analysis

Enriched gene pathways were identified using Ingenuity Pathway Analysis (IPA), the Database for Annotation, Visualization and Integrated Discovery (DAVID), and Gene Set Enrichment Analysis. Significantly regulated genes (*p* < 0.05) were uploaded into IPA software v9.0 (Ingenuity Systems, CA, USA) in separate lists for the combination vs. bicalutamide alone and the combination vs. vorinostat alone. Each gene was mapped to its corresponding molecule in the Ingenuity pathways knowledge base, and core analysis identified enriched pathways and networks in the dataset against a background of the Affymetrix Human GeneChip ST 1.0 array. Lists of genes significantly regulated by the combination compared to either vehicle control or either of the individual agents were uploaded to the Functional Annotation Tool available through DAVID (https://david.ncifcrf.gov/; [29, 30]), converted to DAVID default IDs, and analyzed against a background of the microarray platform. Genes significantly regulated by the combination, either uniquely or when compared with the individual agents, were analyzed against a background of all genes significantly regulated by the combination over vehicle control. Enriched Gene Ontology (GO) biological processes and Kyoto Encyclopedia of Genes and Genomes (KEGG) pathways were identified using DAVID. Gene Set Enrichment Analysis [31] was implemented using the Broad Institute’s public GenePattern server (http://genepattern.broadinstitute.org/gp/pages/index.jsf), with default parameters.

### Transfection of siRNA and expression constructs

For siRNA transfection, LNCaP or VCaP cells were seeded directly into transfection medium containing phenol red free (PRF) RPMI 1640, lipofectamine 2000 (Life Technologies), and reconstituted scrambled siRNA control or siRNA targeting *NFKBIA* at a concentration of 10 nM. Four siRNAs were tested, and #1 and #4 were found to be the most effective at achieving knockdown. After 4 h of culture, additional PRF-RPMI medium containing FBS and L-glutamine was added to the LNCaP transfection mixture, while DMEM containing FBS, L-glutamine and non-essential amino acids was added to the VCaP transfection mixture. Cells were harvested for counting and assessment of cell death using Trypan blue dye exclusion, and then lysed in RIPA buffer for immunoblot analysis three days post-treatment (LNCaP) or six days post-treatment (VCaP). The two timepoints used reflect the different growth kinetics between the two cell lines.

For transient transfection of lentiviral constructs expressing green fluorescent protein (GFP) or co-expressing NFKBIA and GFP, LNCaP cells were seeded at ~40 % confluency and allowed to attach overnight. Growth medium was removed and replaced with transfection medium containing PRF-RPMI, lipofectamine 2000, and 1.5 μg plasmid DNA. As for siRNA transfection, additional medium containing FBS, L-glutamine and either vehicle control or combination therapy was added to the transfection mix after 4 h of culture. At three days post-treatment, fluorescent cells were visualized using a fluorescent microscope, and transfection efficiency was estimated at between 40 and 50 %. Cells were then harvested and assessed for death using Trypan blue dye exclusion, after which they were lysed in RIPA buffer for subsequent immunoblot analysis.

## Results

### The combination of bicalutamide and vorinostat commits AR-dependent prostate cancer cells to death within 8 h of culture

**Fig. 1:**
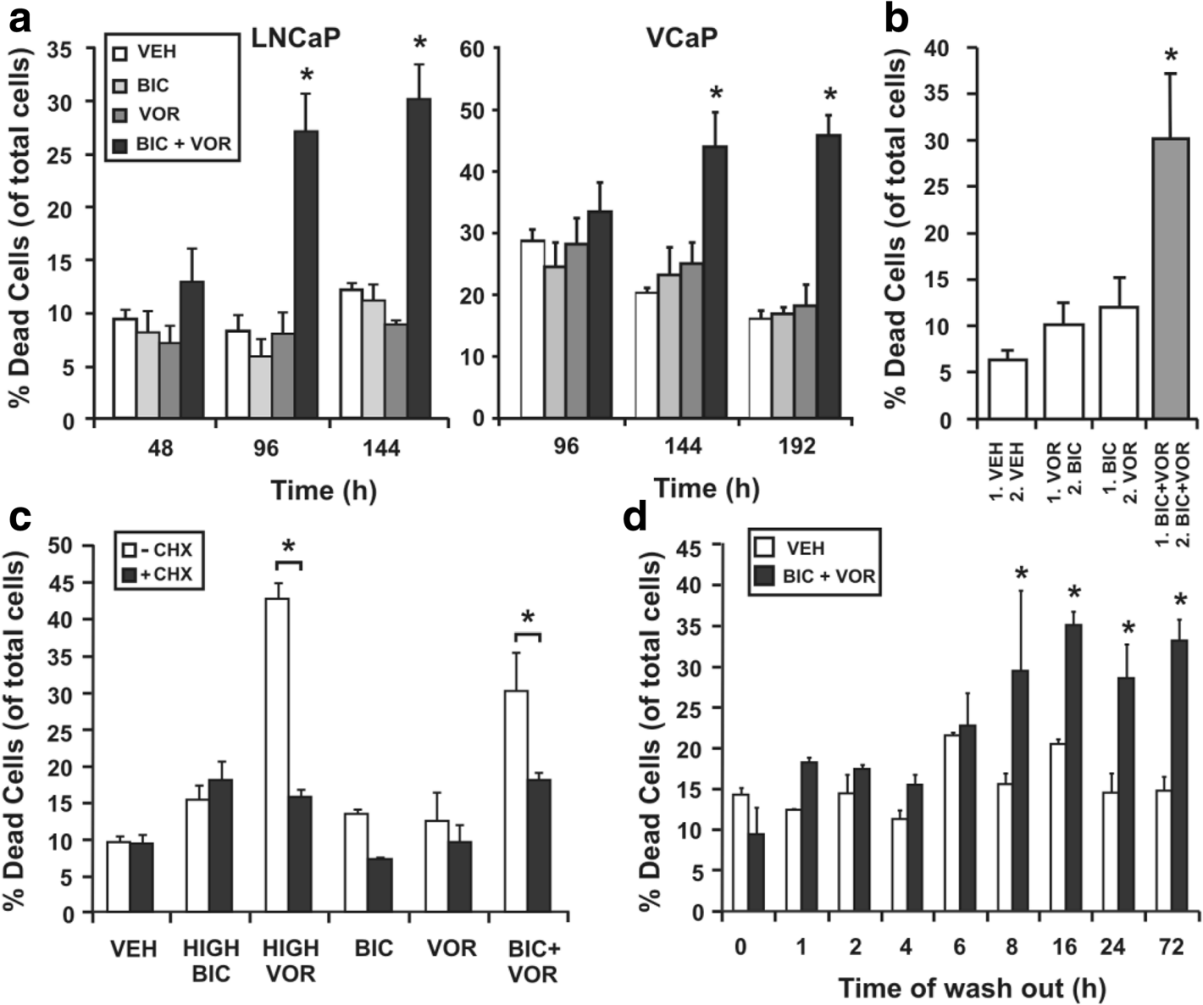
Characterization of cell death caused by the combination of bicalutamide and vorinostat. LNCaP cells (2 × 10^4^ cells per well in 24-well plates) were cultured in triplicate wells with either vehicle control [VEH] bicalutamide [BIC] (2.5 μM) or vorinostat [VOR] (1 μM), individually and in combination [BIC + VOR], in RPMI 1640 supplemented with 10 % FBS. VCaP cells (5 × 10^4^ cells per well in 24-well plates) were cultured in triplicate wells with bicalutamide (1.25 μM) or vorinostat (0.5 μM), individually and in combination, in DMEM supplemented with sodium pyruvate, non-essential amino acids and 10 % FBS. **a** LNCaP cells were counted at 2, 4 and 6 days of culture with bicalutamide and vorinostat, while VCaPs were counted at 4, 6 and 8 days of culture. **b** LNCaP cells were cultured with treatment one (1.) for 24 h, which was removed and replaced with treatment two (2.) for 48 h. **(C)** LNCaP cells were pre-treated for 1 h with cycloheximide (10 μM), and then cultured for 3 d with indicated treatments (HIGH BIC = 50 μM, HIGH VOR = 7.5 μM). **d** LNCaP cells were cultured with indicated treatments, and at 1, 2, 4, 6, 8, 16, or 24 h treatment medium was removed and replaced with normal culture medium not containing either agent until a total of 72 h of culture. At each end point, cells were counted using a haemocytometer, and assessed for viability using trypan blue dye exclusion. Cell death is expressed as a percentage of total cell number. Values indicated are the mean of triplicate wells ± SEM, and are representative of three independent experiments. * = *p* < 0.05 using one-way ANOVA with Bonferroni post-hoc test

Combining the AR antagonist bicalutamide with the HDAC inhibitor vorinostat induces synergistic cell death of prostate cancer cells, with multiple features characteristic of apoptosis [26]. In order to accurately tailor the design of microarray studies, the timing and cellular requirements for cell death induced by the combination were determined in AR-dependent prostate cancer cells. A basal level of cell death of approximately 10 % in LNCaP cells and 15–30 % in VCaP cells was observed when cells were treated with vehicle control and low doses of bicalutamide (2.5 μM) or vorinostat (1 μM) alone (Fig. 1a). A significant increase in cell death was observed in LNCaP (up to 30 %) and VCaP (up to 50 %) cells treated with the combination of bicalutamide and vorinostat (Fig. 1a). To determine whether the synergy was due to one agent sensitizing the cells to the other agent, LNCaP cells were treated sequentially with each individual agent alone and subsequently assessed for viability. Maximal cell death was only achieved when both agents were simultaneously present in culture, not when agents were used sequentially (Fig. 1b). Pre–treatment with the protein synthesis inhibitor cycloheximide indicated that cell death induced by the combination is at least partially reliant on *de novo* protein synthesis. High dose vorinostat, known to rely on *de novo* protein synthesis for induction of cell death [32], was included as a positive control (Fig. 1c). Finally, drug wash out studies determined the timing of molecular events leading to prostate cancer cell death. A percentage of death comparable to a full 3 days of combination therapy (~30 %) was observed when the cells had been cultured with the drugs for at least 8 h or more before drug washout and subsequent culture with standard culture media for 3 days (Fig. 1d), indicating that molecular events that induce irreversible cell death occur within 6–8 h of treatment.

### Gene expression profiles in LNCaP cells treated with bicalutamide and vorinostat

**Fig. 2:**
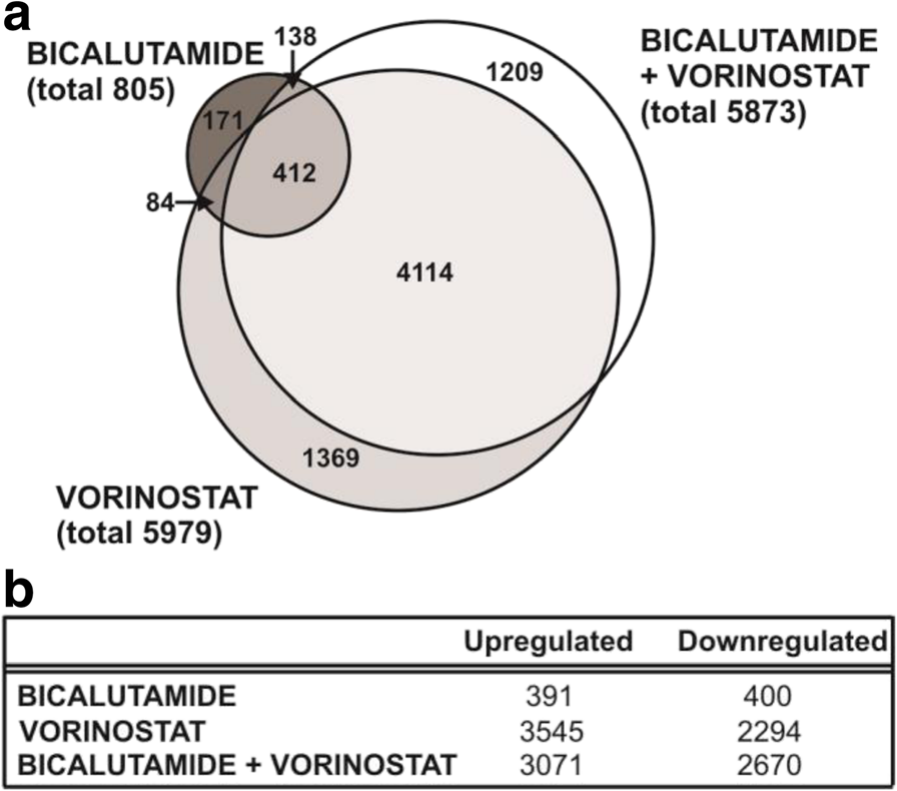
Summary of genes expression changes after treatment with bicalutamide, vorinostat, and the combination. LNCaP cells were treated with vehicle control, bicalutamide, vorinostat and bicalutamide and vorinostat in combination, and RNA was extracted at 6 h of treatment. Microarray analysis was performed on Affymetrix Human GeneChip 1.0 ST Arrays, with six biological replicates per sample. **a** Venn diagram of genes with mRNA levels significantly changed by vorinostat (*light grey*), bicalutamide (*dark grey*) or the doses of vorinostat and bicalutamide in combination (*white*), when compared with vehicle control. *Circles* represent both significantly up-regulated and significantly down-regulated genes over vehicle control, and sizes are proportional to the number of genes. **b** Numbers of genes significantly up-regulated and significantly down-regulated by each treatment when compared with vehicle control

Gene expression profiling of LNCaP cells treated with vehicle control, bicalutamide or vorinostat alone and a combination of the agents was carried out to identify the molecular mechanisms underlying combination-induced cell death. A treatment time of 6 h was selected based on the wash out study (Fig. 1d). A total of 7497 genes were significantly modulated (*p* < 0.05) by the agents alone or in combination when compared with vehicle control (Fig. 2a). Of the 5873 genes modulated by the combination, ~70 % were also modulated by vorinostat alone, ~2 % were also modulated by bicalutamide alone, and ~7 % were modulated by both of the individual agents. A total of 1209 genes (~20 %) were uniquely regulated by the combination. As expected of an HDAC inhibitor, which increases histone acetylation and thereby promotes chromatin accessibility and transcriptional activation, vorinostat induced significantly more genes than it repressed (Fig. 2b). In contrast, bicalutamide alone or the combination induced and repressed approximately equal proportions of genes. Three genes –*PMEPA1*, *PGM2L1* and *STEAP1*- were selected as representative examples of the range of different expression patterns most commonly observed, and quantified by qRT-PCR in the microarray samples and an independently generated sample set. All three genes showed a comparable pattern of regulation in both the microarray and the validation RNA sets (Additional file 3: Figure S2), indicating that the microarray data was robust.

### Combined bicalutamide and vorinostat treatment antagonizes the expression of androgen-regulated genes

**Fig. 3:**
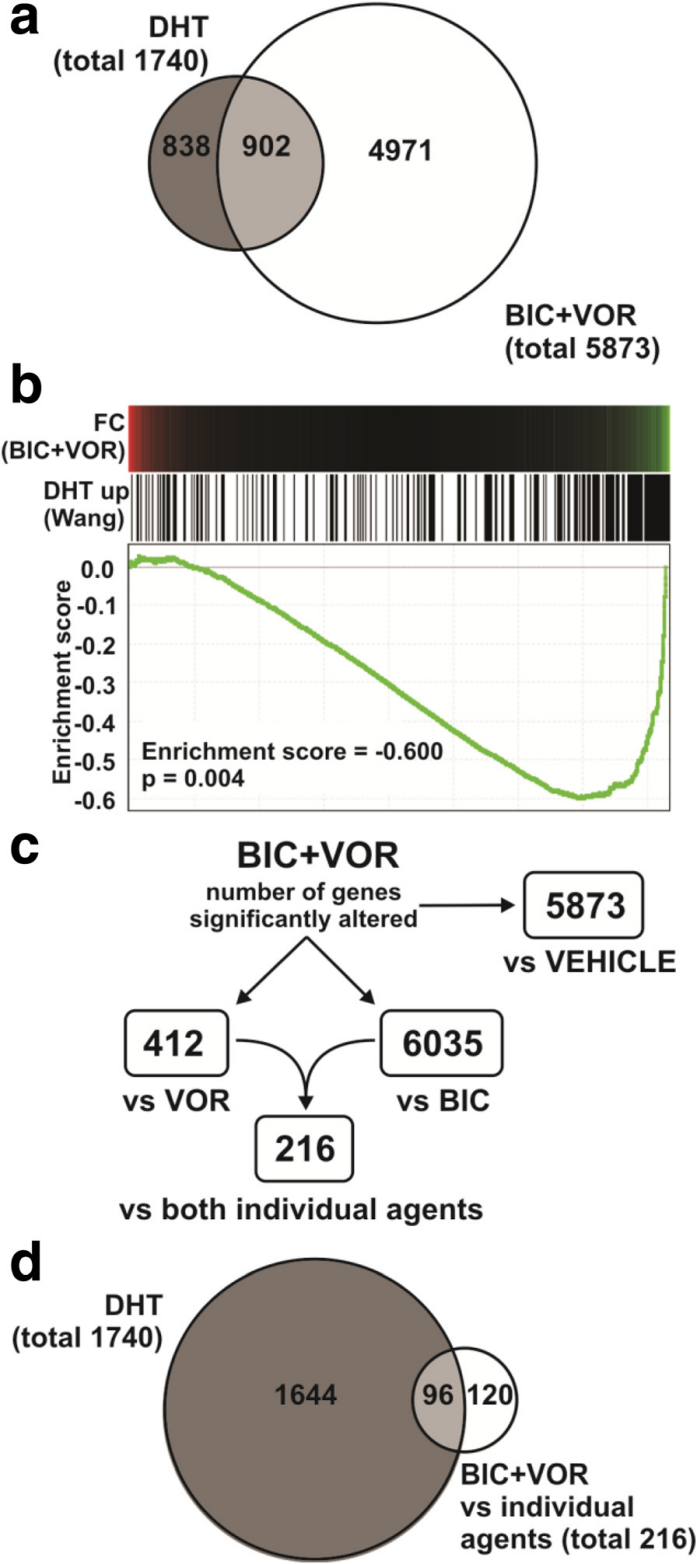
Changes in gene expression induced by bicalutamide and vorinostat in prostate cancer cells are inversely related to androgen-induced gene profiles. **a** Venn diagram of the overlap between genes significantly changed by the combination of bicalutamide and vorinostat compared with vehicle control (*white*) and genes significantly changed by treatment with 100 nM DHT for 16 h from the Wang [33] dataset (*grey*). *Circles* represent both up-regulated and down-regulated genes and are proportional to the number of genes. **b** Genes regulated by treatment with the combination of bicalutamide and vorinostat (compared with vehicle control) are negatively correlated with genes induced by DHT in prostate cancer cells, as assessed by GSEA. Sextuplicate sets of expression profiles were compared between LNCaP cells treated with vehicle control, vorinostat, bicalutamide or the combination of vorinostat and bicalutamide. Probe sets in the data were collapsed to gene level, assigned a score based on a signal-to-noise ratio algorithm and rank-ordered by this score. DHT-induced genes in the ordered data set are shown as *black lines* (*middle*), and the running enrichment score is plotted (*bottom*). The change in expression of each gene in response to the combination treatment is shown as a heatmap (*top*). **c** Flowchart of the refinement process involved in determining genes involved in the cell death caused by the combination. The refined list included the 216 genes that were significantly altered (*p* < 0.05; ANOVA with FDR adjustment) by the combination when compared to both of the individual agents. **d** Venn diagram of genes with mRNA levels significantly changed by the combination of vorinostat and bicalutamide [BIC + VOR vs individual agents] when compared with vehicle control, bicalutamide and vorinostat individually (*white*) and genes regulated by 100 nM DHT from the Wang [33] dataset (*grey*). *Circles* represent both up-regulated and down-regulated genes and are proportional to the number of genes

As both bicalutamide and vorinostat have been shown to modulate AR levels and activity, it was feasible that cell death induced by the combination results from enhanced blockade of androgen signaling and the consequent antagonism of androgen-regulated genes required for growth and survival. To assess this possibility, genes modulated by the combination therapy were compared with LNCaP androgen-regulated genes defined in a previous study by Wang and colleagues [33]. Approximately half of the androgen-regulated genes in LNCaP cells were significantly altered by the combination treatment (Fig. 3a). As expected, gene set enrichment analysis (GSEA) revealed that the genes upregulated by androgen treatment were generally repressed by the combination treatment (Fig. 3b).

**Fig. 4:**
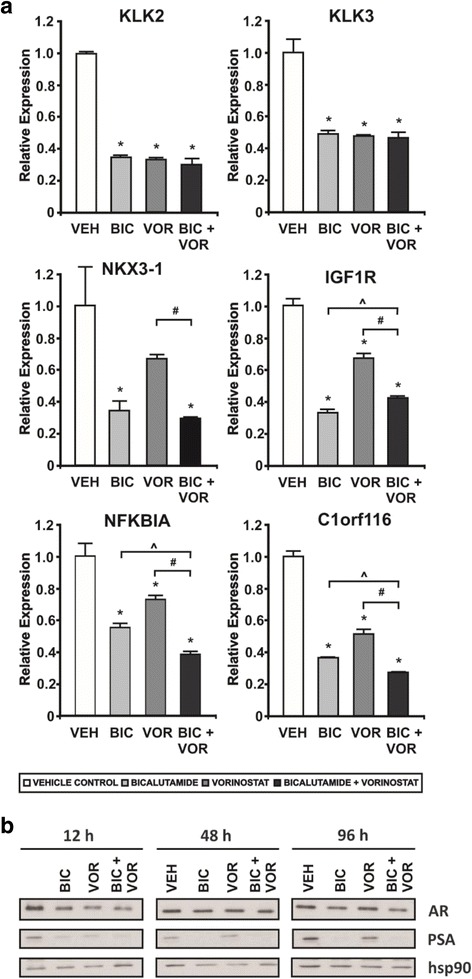
Quantitative analysis of androgen regulated genes altered by combinatorial AR targeting. **a** Quantitative real-time PCR analysis of an independent RNA sample set, generated by treatment of LNCaP cells with vehicle control [VEH], 1 μM vorinostat [VOR], 2.5 μM bicalutamide [BIC] or the combination of 1 μM vorinostat and 2.5 μM bicalutamide [BIC + VOR] in triplicate for 6 h. The expression of *KLK2*, *KLK3*, *NKX3-1*, *IGF1R*, *NFKBIA* and *C1orf116* was normalised to *GUSB* and *HPRT1*. Fold changes are expressed relative to vehicle control. Values indicated are the mean of technical and biological replicates ± SEM, and are representative of three independent experiments. * = *p* < 0.05 using one-way ANOVA with Bonferroni post-hoc test, compared with vehicle control. # = *p* < 0.05 using *t*-test compared with vorinostat, and ^ = *p* < 0.05 using *t*-test compared with bicalutamide. **b** Western blot analysis of lysates from LNCaP cells cultured with either vehicle control [VEH], 1 μM vorinostat [VOR], 2.5 μM bicalutamide [BIC] or the combination of 1 μM vorinostat and 2.5 μM bicalutamide [BIC+VOR] for 12, 48, or 96 h. Steady state levels of PSA (*KLK3*) are shown, and hsp90 is shown as a loading control

A high proportion of the genes significantly regulated by the combination were also regulated to a similar magnitude by bicalutamide or vorinostat treatment alone. However, as induction of cell death was restricted to the combination treatment, we focused on the genes that showed selective alteration with the combination compared to the individual agents. Combination treatment altered 412 genes compared to vorinostat alone and 6035 genes compared to bicalutamide alone. We refined these lists to include only the genes that were significantly altered (*p* < 0.05; ANOVA with FDR adjustment) by the combination when compared to both of the individual agents, which yielded 216 genes (Fig. 3c; full list shown in Additional file 4: Table S2). Interestingly, approximately half of the genes in this set have been reported to be androgen-regulated (Fig. 3d), suggesting that enhanced blockade of androgen signaling by the combination was likely to be a mechanism of cell death. To test this hypothesis, six known androgen regulated genes, *KLK2*, *KLK3* (PSA), *NKX3-1*, *IGF1R*, *NFKBIA* and *C1orf116*, that exhibited significantly greater regulation by the combination in the microarray compared with individual agents alone were analyzed by qRT-PCR in an independent sample set. Each of these genes are frequently used to assess androgen receptor signaling (32). All six genes were significantly down regulated (>2-fold) after 6 h of treatment with the combination when compared with vehicle control (Fig. 4a). For *KLK2*, *KLK3* (PSA), *NKX3-1*, *IGF1R*, and *C1orf116*, the effect of the combination therapy largely paralleled that of bicalutamide treatment alone; comparable activity of both treatments was also observed at the protein level for PSA (*KLK3*) (Fig. 4b). Interestingly, *NFKBIA*, an androgen regulated gene that encodes an inhibitor of the NF-κB complex (IκBα), was significantly and consistently down regulated by the combination compared to individual treatments of both bicalutamide and vorinostat at 6 h of culture. Collectively, these observations suggested that prostate cancer cell death induced by the combination may not be occurring solely due to more complete blockade of androgen signaling, and other pathways involving a subset of androgen-regulated and non-androgen-regulated genes are likely to be involved.

### Loss of *NFKBIA* contributes to cell death induced by the combination of bicalutamide and vorinostat

To investigate other cellular pathways that potentially mediate the effects of the combination therapy, three independent gene annotation enrichment programs were utilized: Ingenuity Pathway Analysis (IPA), the Database for Annotation, Visualization and Integrated Discovery (DAVID), and GSEA. Due to the significant overlap between pathways enriched by the combination and the individual agents (Additional file 5: Table S3), we restricted our analysis to the set of 216 genes (Fig. 3c) that were significantly regulated by the combination treatment when compared to the effects of the individual agent treatments. This refined gene set was enriched for a number of pathways potentially important in the synergistic action of the combination therapy, including pathways involved in cell cycle, cell death and cell motility, such as p53 signaling, Jak-STAT signaling, and axon guidance (Table 1).

**Table 1:** Pathway analysis for genes significantly modulated by the combination compared to individual agents alone

Ingenuity pathway analysis			DAVID (KEGG) pathway analysis		
Pathway ID	Genes involved	*p*-value	Pathway ID	Genes involved	*p*-value
Molecular Mechanisms of Cancer	FYN, PMAIP1, NFKBIA, GNA15, RHOU, PLCB1	8.91 × 10^−7^	Pathways in cancer	IGF1R, LAMA1, LAMA3, KLK3, NKX3-1, NFKBIA, ZBTB16, PIAS1, MMP1	1.01 × 10^−8^
Inositol Phosphate Metabolism	SYNJ1, SGK1, PLCB1, MAK	2.75 × 10^−6^	Focal adhesion	IGF1R, LAMA1, LAMA3, FYN, THBS1, VCL	2.66 × 10^−6^
p53 Signaling	PMAIP1, TP53INP1, THBS1, SNAI2, TNFRSF10B	3.16 × 10^−6^	Jak-STAT signaling pathway	SOCS2, LIFR, PIAS1, IL6R	2.48 × 10^−4^
Prostate Cancer Signaling	NFKBIA, NKX3-1, KLK3	8.13 × 10^−5^	Small cell lung cancer	LAMA1, LAMA3, NFKBIA, PIAS1	2.84 × 10^−4^
Aldosterone Signaling in Epithelial Cells	SGK1, PLCB1, DNAJB14	2.88 × 10^−4^	Prostate cancer	IGF1R, KLK3, NKX3-1, NFKBIA	3.02 × 10^−4^
Glioma Signaling	IGF1R	3.63 × 10^−4^	Adherens junction	IGF1R, FYN, SNAI2, VCL	3.22 × 10^−4^
Biosynthesis of Steroids	HMGCR	5.25 × 10^−4^	Axon guidance	NRP1, FYN, NTNG1, EFNA5	4.08 × 10^−4^
NRF2-mediated Oxidative Stress Response	GSTM2, MAF, DNAJB14, ABCC4	0.0010	ECM-receptor interaction	LAMA1, LAMA3, THBS1	0.0018
Xenobiotic Metabolism Signaling	GSTM2, MAF, CYP3A5	0.0013	Long-term depression	IGF1R, GUCY1A3, PLCB1	0.0029
TWEAK Signaling	NFKBIA	0.0016	Adipocytokine signaling pathway	NFKBIA, ACSL3, CAMKK2	0.0038
TNFR1 Signaling	NFKBIA	0.0018	Gap junction	TUBB, GUCY1A3, PLCB1	0.0047
TNFR2 Signaling	NFKBIA	0.0023	p53 signaling pathway	TNFRSF10B, PMAIP1, THBS1	0.0064
Role of CHK Proteins in Cell Cycle Checkpoint Control	HUS1	0.0026	T cell receptor signaling pathway	CD8B, FYN, NFKBIA	0.0073
B Cell Receptor Signaling	NFKBIA	0.0037	Purine metabolism	POLE2, GUCY1A3, PDE9A	0.0095
Prolactin Signaling	FYN, SOCS2	0.0038	Cytokine-cytokine receptor interaction	TNFRSF10B, LIFR, IL6R	0.0103
PI3K/AKT Signaling	NFKBIA	0.0060	Hematopoietic cell lineage	CD8B, IL6R	0.0355
Insulin Receptor Signaling	FYN, SGK1	0.0065	ABC transporters	ABCC4, ABCG1	0.0462
Death Receptor Signaling	NFKBIA, TNFRSF10B	0.0083	Drug metabolism	CYP3A5, NAT1	0.0462

Interestingly, the androgen-regulated gene *NFKBIA* (nuclear factor of kappa light polypeptide gene enhancer in B-cells inhibitor, alpha) was present in many of these pathways, implying that it may be a key regulator of the combination therapy. Supporting this concept, the network tool in IPA identified *NFKBIA* or its associated genes as central molecules in the top two significantly enriched networks - Cell Morphology, Cellular Movement and Cell Signaling, and Cellular Movement, Drug Metabolism, Endocrine System Development and Function (Additional file 6: Figure S3, Additional file 7: Table S4). Furthermore, when scrutinizing the list of 216 genes with enhanced regulation by the combination, *NFKBIA* was one of the most markedly down-regulated genes in response to combination treatment (>1.5 fold change) when compared to both of the individual agents alone (Additional file 4: Table S2). These results collectively implicated a role for *NFKBIA* in the molecular action of the combination treatment.

**Fig. 5:**
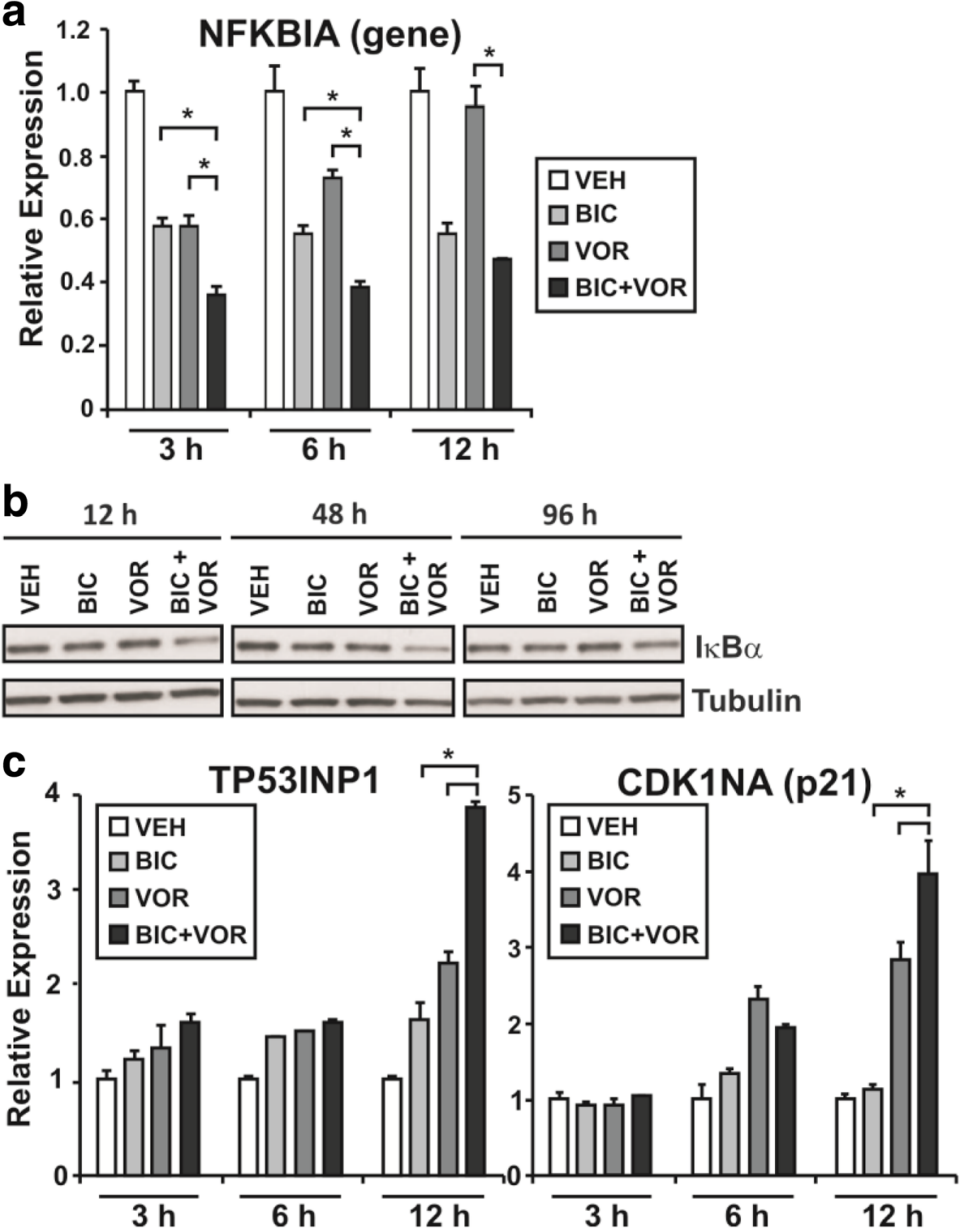
The effect of combinatorial AR targeting on *NFKBIA* levels and p53 signaling. **a** The expression of *NFKBIA* over a timecourse was normalized to *GUSB* and *HPRT1*. Relative expression is the fold change expressed relative to vehicle control. Values indicated are the mean of technical and biological replicates ± SEM, and are representative of three independent experiments. * = *p* < 0.05 using one-way ANOVA with Bonferroni post-hoc test, compared with bicalutamide or vorinostat individually. **b** Western blot analysis of lysates from LNCaP cells cultured with either vehicle control [VEH], 1 μM vorinostat [VOR], 2.5 μM bicalutamide [BIC] or the combination of 1 μM vorinostat and 2.5 μM bicalutamide [BIC+VOR] for 12, 48, or 96 h. Steady state levels of IκBα are shown, and tubulin is shown as a loading control. **c** mRNA expression levels of *TP53INP1* and *CDKN1A* are shown as for *NFKBIA*. * = *p* < 0.05 using one-way ANOVA with Bonferroni post-hoc test

Down-regulation of *NFKBIA* by the combination treatment was validated in a set of RNA samples generated from an independent time-course experiment (Fig. 5a). *NFKBIA* mRNA was repressed within 3 h by the combination compared to both vehicle (approximately 4-fold down-regulated) and to each agent individually (approximately 2-fold). This repression was maintained for at least 6 h of culture, but returned to levels similar to bicalutamide alone by 12 h. Western blot analysis showed that protein levels of the corresponding protein, IκBα, were also reduced by the combination (Fig. 5b).

The significant and rapid modulation of *NFKBIA* suggested that it might be an upstream initiator of processes involved in cell death mediated by the combination therapy. *NFKBIA* is a known regulator of the NF-κB and p53 signaling pathways [34], and p53 signaling was identified as a highly enriched pathway in cells treated with the combination (Table 1). Given this association between *NFKBIA* (IκBα) and p53 signaling, the expression of two p53 inducible genes, *TP53INP1* and *CDKN1A* (p21) was measured. At early time points of 3 and 6 h, neither *TP53INP1* nor *CDKN1A* mRNA was altered by combination therapy or either of the agents individually. However, at 12 h of culture, the combination induced expression of both genes by approximately four-fold when compared with vehicle control, and two-fold when compared with bicalutamide or vorinostat (Fig. 5c). The up-regulation of *TP53INP1* and *CDKN1A* occurred after the decrease in *NFKBIA*, and at a similar time point to the observed loss of IκBα protein (Fig. 5b), supporting the hypothesis that the combination mediates *NFKBIA*-dependent activation of p53 signaling.

### Specific knockdown or overexpression of NFKBIA alters LNCaP cell viability and response to the combination of bicalutamide and vorinostat

**Fig. 6:**
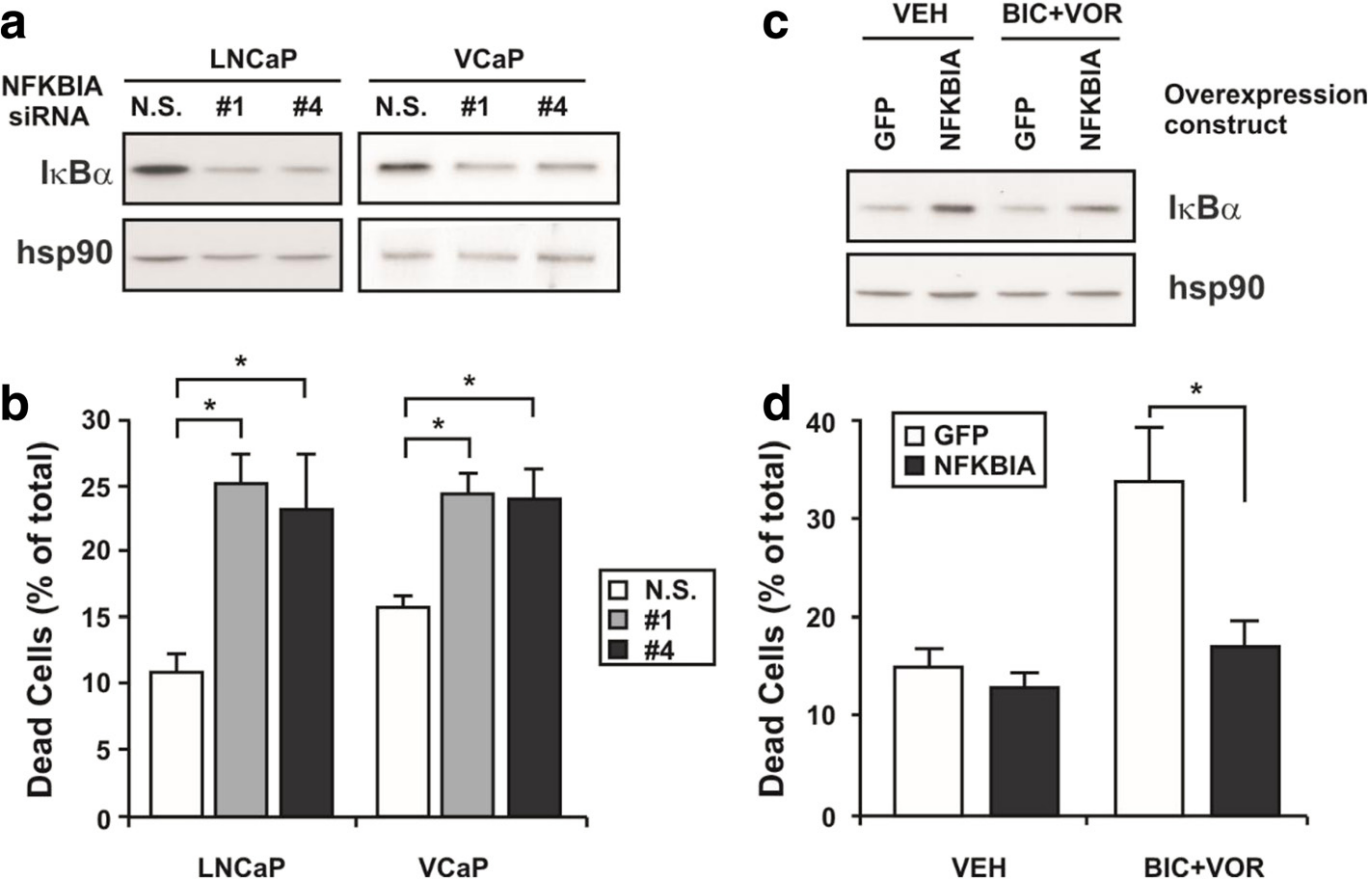
*NFKBIA* is a critical mediator of death induced by combinatorial AR targeting. LNCaP cells (1 × 10^5^ cells per well in 12-well plates) or VCaP cells (2 × 10^5^ cells per well in 12-well plates) were transfected in triplicate wells with 10 nM of either non-specific, scrambled siRNA [N.S.] or specific siRNAs [siRNA #1 and siRNA #4] targeting *NFKBIA*. LNCaP cells were transfected in triplicate wells with 1.5 μg of plasmid expressing either GFP or NFKBIA+GFP, for 4 h. The transfection medium was then overlaid with vehicle control [VEH], or the combination [BIC + VOR] to give a final concentration of 2.5 μM bicalutamide and 1 μM vorinostat in either PRF-RPMI 1640 supplemented with 10 % FBS and 1 % l-glutamine (LNCaP) or DMEM supplemented with non-essential amino acids, 1 % l-glutamine and 10 % FBS (VCaP). **a** Western blot analysis of lysates from LNCaP and VCaP cells transfected with either non-specific [N.S.] or specific [#1 and #4] *NFKBIA* siRNA. Steady state levels of IκBα at day three (LNCaP) or day six (VCaP) of treatment are shown, with hsp90 as a loading control. **b** Cells were counted at 3 days (LNCaP) or 6 days (VCaP) of treatment, and assessed for viability using trypan blue dye exclusion. Cell death is expressed as a percentage of total cell number. Values indicated are the mean of triplicate wells ± SEM, and are representative of three independent experiments. **c** Western blot analysis of lysates from LNCaP cells transfected with plasmids expressing GFP only [GFP] or NFKBIA and GFP [NFKBIA], and then treated with vehicle control [VC] or combination [BIC+VOR]. Steady state levels of IκBα at day three of treatment are shown, with hsp90 as a loading control. **d** Cells were counted at 3 days of treatment, and assessed for viability using trypan blue dye exclusion. Cell death is expressed as a percentage of total cell number. Values indicated are the mean of triplicate wells ± SEM, and are representative of three independent experiments. * = *p* < 0.05 using *t*-test

To investigate the functional significance of *NFKBIA* for prostate cancer cell viability and the observed drug responses, siRNA-mediated knockdown of *NFKBIA* was performed in both LNCaP and VCaP cells. Transfection with two different siRNAs targeting *NFKBIA* resulted in a marked reduction in IκBα protein levels at 3 days post-treatment for LNCaPs, and 6 days post-treatment for VCaPs (Fig. 6a). Depletion of IκBα with siRNA #1 or #4 caused a significant induction of cell death in LNCaP cells (23–25 % compared to the basal level of 10 % observed with non-specific siRNA; *p* < 0.05; Fig. 6b) and VCaP cells (25 % compared to the basal level of 15 % observed with non-specific siRNA; *p* < 0.05; Fig. 6b). This data indicated that IκBα is an important regulator of prostate cancer cell viability in two separate AR-positive cell lines. Moreover, IκBα depletion caused cell death of a similar magnitude to that observed with combination treatment, and significantly enhanced cell death caused by the combination (Additional file 8: Figure S4).

To directly assess the importance of IκBα down-regulation in combination therapy-mediated cell death, we tested whether ectopic over-expression of *NFKBIA* altered response to the combination treatment. Transfection efficiency of the *NFKBIA* expression construct, which co-expresses GFP, was estimated at between 30 and 50 % by flow cytometry and fluorescent microscopy (data not shown). Increased levels of IκBα protein after transfection, compared to cells transfected with a control GFP-only expression vector, were confirmed by immunoblotting (Fig. 6c). Treatment of cells overexpressing GFP with the combination of vorinostat and bicalutamide induced approximately 34 % cell death which, as expected, was significantly greater than in vehicle-treated controls. By contrast, in cells overexpressing IκBα, cell death caused by the combination was significantly reduced to ~17 %, which was not significantly different to the basal cell death observed with vehicle control treatment. This finding indicates that downregulation of IκBα is an essential requirement for synergistic cell death induced by the combination of bicalutamide and vorinostat.

## Discussion

Metastatic prostate cancer inevitably becomes resistant to current hormonal therapies and, consequently, combinatorial therapeutic approaches that may be more efficacious and less prone to resistance-associated failure have garnered significant interest. We have previously shown that combining the AR antagonist bicalutamide with the HDAC inhibitor vorinostat synergistically induces growth arrest and cell death in prostate cancer cells. Importantly, this combination approach uses doses of both agents that are individually sub-effective [26], implying that it would minimize dose-related toxicity. The current study provides new insight into the molecular mechanism by which these disparate agents interact synergistically to induce death of prostate cancer cells, and implicates IκBα, a regulator of the NF-κB and p53 pathways, as a critical factor for prostate cancer cell viability and treatment response.

Given that vorinostat and bicalutamide both target AR, we initially hypothesized that the combination of these two agents would enhance blockade of androgen signaling, a pathway that promotes growth and survival of prostate cancer cells. This hypothesis was reinforced by previous work from our laboratory demonstrating that the combination treatment induced death only in cells with a functional AR signaling axis, and that addition of excess dihydrotestosterone (DHT) to the system prevented cell death [26]. The genome-wide microarray expression data generated in the current study also supported this hypothesis. Specifically, many androgen-regulated genes were significantly altered by the combination compared to individual agents, and pathway analysis demonstrated deregulation of androgen signaling and prostate cancer networks by the combination treatment. Interestingly, for six known androgen regulated genes, we did not observe consistently greater downregulation by the combination therapy in an independent set of RNA samples. This could suggest that enhanced androgen blockade is not the substantive mechanism by which the combination exerts its effect. However, this subset of genes only represents approximately 10 % of the genes significantly regulated by both DHT and the combination (when compared with the individual agents), and 1 % of the total genes significantly regulated by both DHT and the combination (when compared with vehicle control). It is possible that small changes to a large number of androgen regulated genes is an important factor in the mechanism of action of the combination therapy, or that these cumulative changes are able to sensitize the prostate cancer cells to HDAC inhibition.

While the importance of enhanced blockade of androgen signaling by the combination treatment remains ambiguous, the expression data revealed that this treatment also modulates a multitude of other critical cellular processes. For example, p53 signaling and other pathways involved in cell cycle arrest and cell death were highly enriched in genes modulated by the combination. In considering potential mediators of the synergistic interaction between vorinostat and bicalutamide, we noted that our pathway analyses consistently implicated an inhibitor of NF-κB signaling, *NFKBIA*, in this phenomenon. Interestingly, *NFKBIA* is an androgen regulated gene, and the protein encoded by this gene, IκBα, is best known as an inhibitor of NF-κB signaling. However, IκBα can also inhibit p53 signaling and thereby functions dichotomously to either block p53-mediated cell death or NF-κB-mediated cell growth [34–37], with the final phenotypic outcome likely depending on the relative levels of NF-κB and p53 within a given cell. At a mechanistic level, IκBα sequesters NF-κB or p53 in the cytoplasm in an inactive complex; following various stimulatory events, IκBα is phosphorylated and targeted for degradation via the ubiquitin-proteasome pathway, relieving inhibition of these factors. With respect to prostate cancer cell death, we observed rapid downregulation of *NFKBIA* in cells treated with the combination of bicalutamide and vorinostat that was associated with increased expression of *TP53INP1 and CDKN1A*, two commonly known p53-inducible genes, and induction of cell death. Taken together, this data suggests that, in the context of the combination therapy, loss of IκBα results in cell death, which may be facilitated by the induction of p53 signaling. This hypothesis is supported by the observation that knockdown of *NFKBIA* in the absence of drug treatment resulted in a similar level of cell death to that observed with the combination in two independent prostate cancer cell lines. Moreover, co-treatment with *NFKBIA* siRNA and the combination therapy caused significantly more cell death than either individual treatment. Importantly, overexpression of NFKBIA almost completely negated the effect of the combination treatment on cell death.

Two other observations arising from the current study are worth noting for their clinical ramifications. First, the combination of bicalutamide and vorinostat was efficacious in models with a mutant (LNCaP) or amplified wild-type (VCaP) AR gene. Given that many clinical prostate cancers are characterized by aberrant AR signaling, and that intra-tumoral heterogeneity may result in foci that each potentially have structurally different androgen receptors, this is a promising feature of the combination therapy. Second, both vorinostat and bicalutamide are required simultaneously in culture for induction of cell death, indicating that if sensitization is happening it occurs rapidly. This finding indicates that future clinical testing will require the agents to be dosed together and not sequentially.

## Conclusion

In summary, we have defined a novel mechanism of action by which bicalutamide and vorinostat, when used in combination, mediate death of prostate cancer cells. While enhanced blockade of androgen signaling is potentially important, we have demonstrated that other cellular pathways also play critical roles. Specifically, IκBα was identified as a critical regulator of therapy-mediated cell death; this factor may have potential either as a new therapeutic target and/or a marker of drug response. The ability to monitor molecular markers of apoptotic response to such therapeutic strategies will aid in the clinical development of this combinatorial approach for treatment of prostate cancer.

## Additional files

Additional file 1: Figure S1. Dose response curves of bicalutamide and vorinostat in VCaP cells. VCaP cells (5 × 10^4^ cells per well in 24-well plates) were cultured in triplicate wells with bicalutamide or vorinostat at the indicated doses in DMEM medium supplemented with sodium pyruvate, non-essential amino acids and 10 % FBS. Cells were counted using a haemocytometer, and assessed for viability using trypan blue dye exclusion. Cell death is expressed as a percentage of total cell number. (PPTX 216 kb) Additional file 2: Table S1. Primer Sequences. (DOCX 13 kb) Additional file 3: Figure S2. Microarray validation by RT-PCR. Validation of the microarray results, using three of the most markedly changed genes: *PMEPA1* was down-regulated by all three treatments, *PGM2L1* was up-regulated by vorinostat and the combination but not bicalutamide, and *STEAP1* was down-regulated uniquely by the combination. Expression values taken from the microarray, qRT-PCR on three of the biological replicates used in the microarray, and qRT-PCR on an independently generated sample set were compared for these three genes. Three technical replicates for each biological replicate were analysed, and the expression normalised to *RPL32* and *GUSB*. Fold change was determined over vehicle control. Values indicated are the mean of technical and biological replicates ± SEM. *p* < 0.05 using one-way ANOVA with Tukey post-hoc test, compared with vehicle control (*), vorinostat (#), or bicalutamide (^). (TIF 6450 kb) Additional file 4: Table S2. List of 216 genes significantly regulated by combination therapy versus each individual agent. (DOCX 28 kb) Additional file 5: Table S3. Comparison of pathway analyses for bicalutamide, vorinostat, or the combination versus vehicle control. KEGG pathways. (DOCX 24 kb) Additional file 6: Figure S3 Enriched networks in list of 216 genes with enhanced regulation by the combination. The network tool in Ingenuity Pathway Analysis (IPA) identified the networks of Cell Morphology, Cellular Movement and Cell Signaling **(A)**, as well as Cellular Movement, Drug Metabolism, Endocrine System Development and Function **(B)**, as the two most significantly enriched by the combination treatment when compared to the individual agents (list of 216 genes). Colors indicate the regulation by the combination when compared to both of the individual agents – green is downregulation, red is upregulation and half green half red means that the combination upregulated the gene compared to one treatment, and downregulated compared to the other. The lines in between the genes represent a network connection. (PPTX 18374 kb) Additional file 7: Table S4. IPA network analysis for genes regulated by the combination when compared to individual doses of bicalutamide and vorinostat. (DOCX 18 kb) Additional file 8: Figure S4. Knockdown of *NFKBIA* enhances the cell death effect observed with the combination therapy. **(A)** Western blot analysis of lysates from LNCaP cells transfected with either non-specific [N.S.] or specific [siRNA #1 and siRNA #4] *NFKBIA* siRNA and then treated with vehicle control [VC], 2.5 μM bicalutamide [BIC], 1 μM vorinostat [VOR], or the combination of 2.5 μM bicalutamide and 1 μM vorinostat [BIC + VOR]. Steady state levels of IκBα at day three of treatment are shown, with hsp90 as a loading control. **(B)** Cells were counted at three days of treatment, and assessed for viability using trypan blue dye exclusion. Cell death is expressed as a percentage of total cell number. Values indicated are the mean of triplicate wells ± SEM, and are representative of three independent experiments. * = *p* < 0.05 using *t*-test. (PPTX 213 kb)

## Abbreviations

ADT: androgen deprivation therapy
AR: androgen receptor
CRPC: castration-resistant prostate cancer
DAVID: database for annotation, visualization and integrated discovery
DHT: 5α-dihydrotestosterone
FBS: fetal bovine serum
FDR: false discovery rate
GFP: green fluorescent protein
GO: gene ontology
GSEA: gene set enrichment analysis
HDAC: histone deacetylase
HSP90: heat shock protein 90
IPA: ingenuity pathway analysis
KEGG: Kyoto encyclopedia of genes and genomes
NFKBIA: nuclear factor of kappa light polypeptide gene enhancer in B-cells inhibitor, alpha
PRF: phenol red-free
PSA: prostate-specific antigen
