# Erratum to: IκBα mediates prostate cancer cell death induced by combinatorial targeting of the androgen receptor

**DOI:** 10.1186/s12885-016-2215-3

**Published:** 2016-03-03

**Authors:** Sarah L. Carter, Margaret M. Centenera, Wayne D. Tilley, Luke A. Selth, Lisa M. Butler

**Affiliations:** Dame Roma Mitchell Cancer Research Laboratories, Adelaide Prostate Cancer Research Centre and Freemason’s Foundation Centre for Men’s Health, School of Medicine, University of Adelaide and Hanson Institute, Adelaide, SA 5005 Australia; Cancer Theme, South Australian Health and Medical Research Institute, Adelaide, SA 5001 Australia

## Erratum

After publication of the original article [1], the authors requested a change in name format: for the middle name initials, and not the full author names, to be used. This has been changed here and in the original manuscript.

## References

[1] Carter SL, Centenera MM, Tilley WD, Selth LA, Butler LM (2016). IκBα mediates prostate cancer cell death induced by combinatorial targeting of the androgen receptor. BMC Cancer..

