# Supplementary figures and images for: IκBα mediates prostate cancer cell death induced by combinatorial targeting of the androgen receptor
Source: BMC Cancer. 2016 Feb 23;16:141. doi: 10.1186/s12885-016-2188-2 (PMC4785192; doi:10.1186/s12885-016-2188-2)

## Slide 1
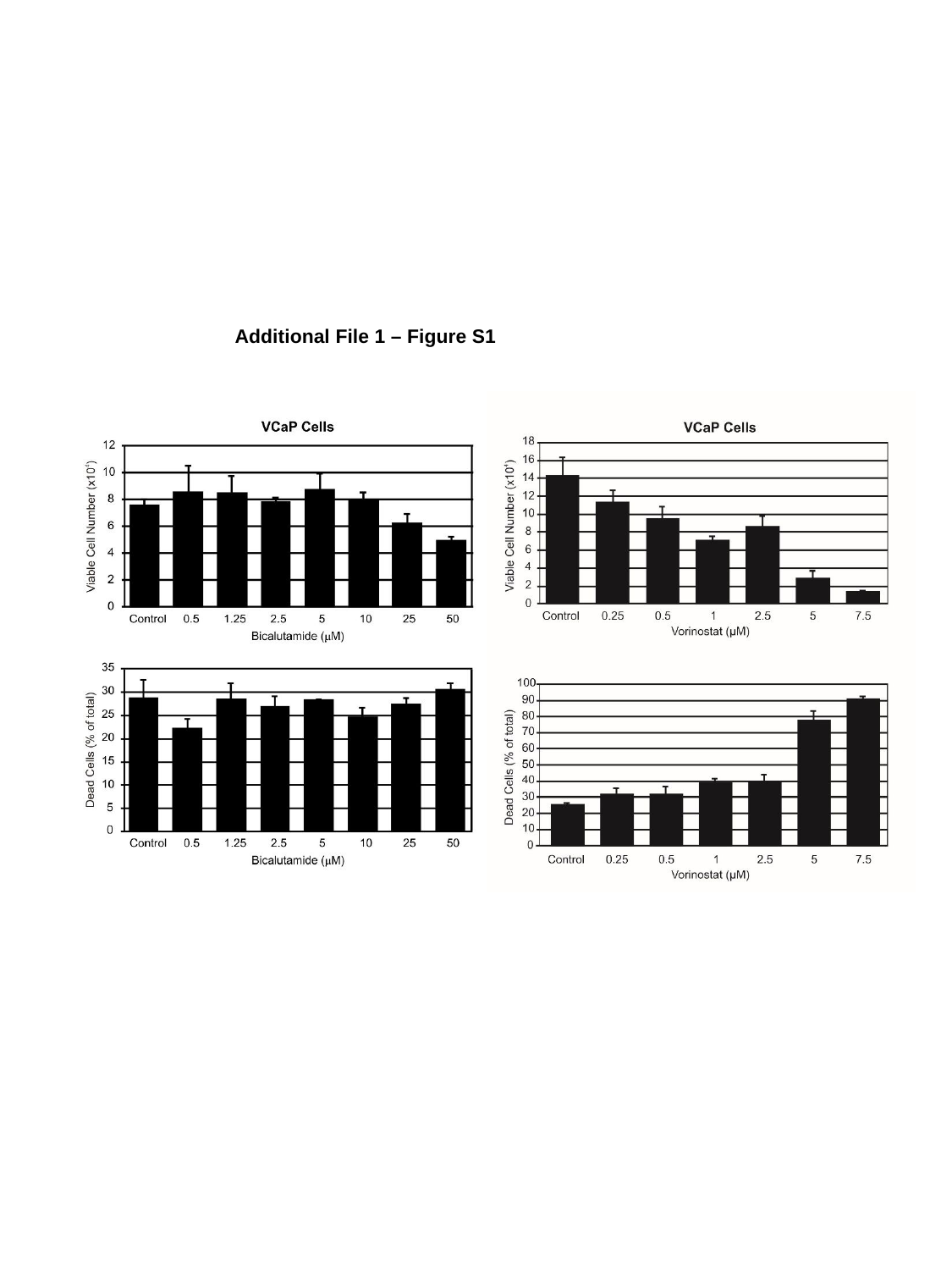

Additional File 1 – Figure S1

Supplement: Additional file 1: Figure S1. — Dose response curves of bicalutamide and vorinostat in VCaP cells. VCaP cells (5 × 10^4^ cells per well in 24-well plates) were cultured in triplicate wells with bicalutamide or vorinostat at the indicated doses in DMEM medium supplemented with sodium pyruvate, non-essential amino acids and 10 % FBS. Cells were counted using a haemocytometer, and assessed for viability using trypan blue dye exclusion. Cell death is expressed as a percentage of total cell number. (PPTX 216 kb) [file 12885_2016_2188_MOESM1_ESM.pptx]

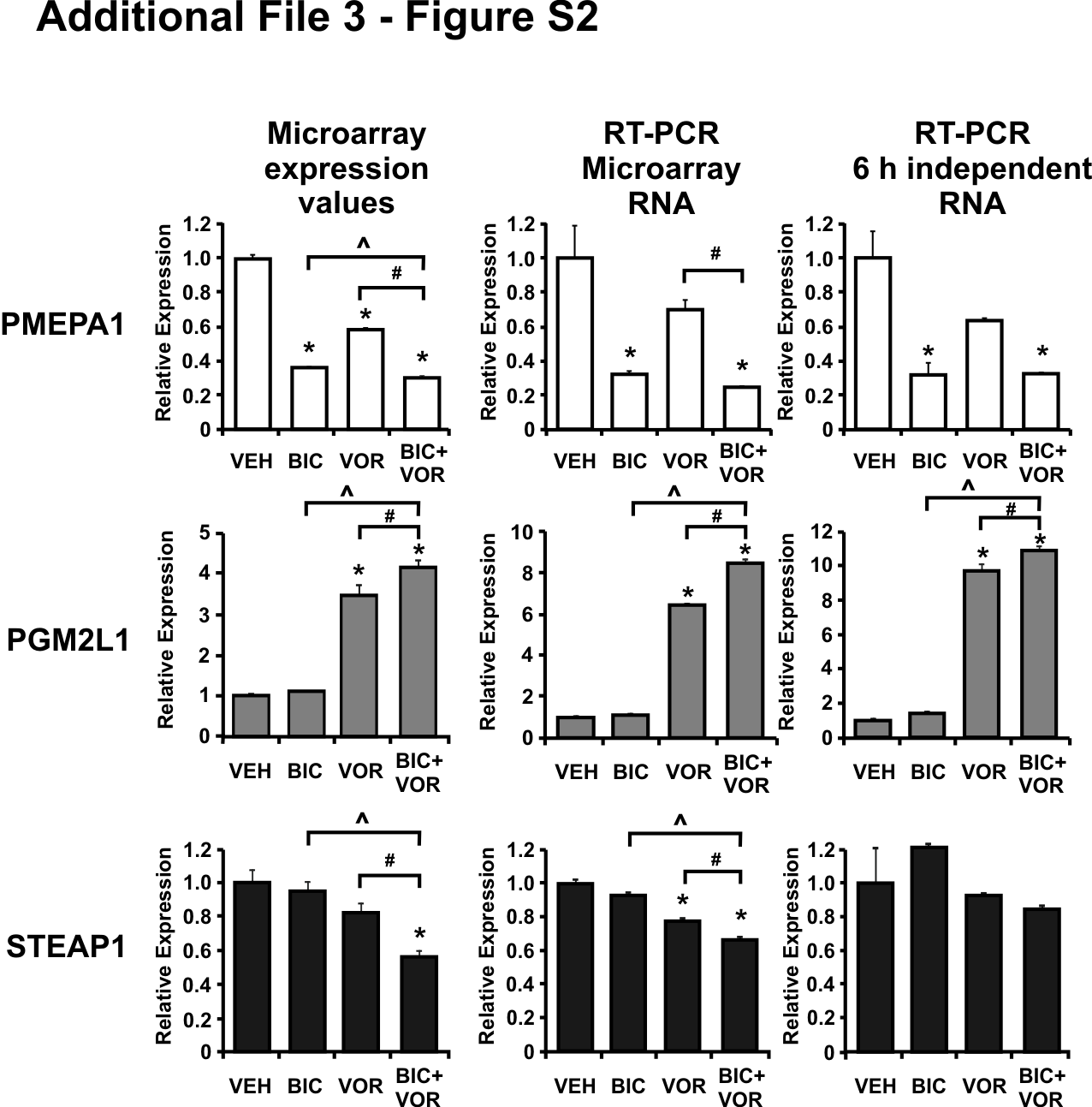

Supplement: Additional file 3: Figure S2. — Microarray validation by RT-PCR. Validation of the microarray results, using three of the most markedly changed genes: PMEPA1 was down-regulated by all three treatments, PGM2L1 was up-regulated by vorinostat and the combination but not bicalutamide, and STEAP1 was down-regulated uniquely by the combination. Expression values taken from the microarray, qRT-PCR on three of the biological replicates used in the microarray, and qRT-PCR on an independently generated sample set were compared for these three genes. Three technical replicates for each biological replicate were analysed, and the expression normalised to RPL32 and GUSB. Fold change was determined over vehicle control. Values indicated are the mean of technical and biological replicates ± SEM. p < 0.05 using one-way ANOVA with Tukey post-hoc test, compared with vehicle control (*), vorinostat (#), or bicalutamide (^). (TIF 6450 kb) [file 12885_2016_2188_MOESM3_ESM.tif]

## Slide 1
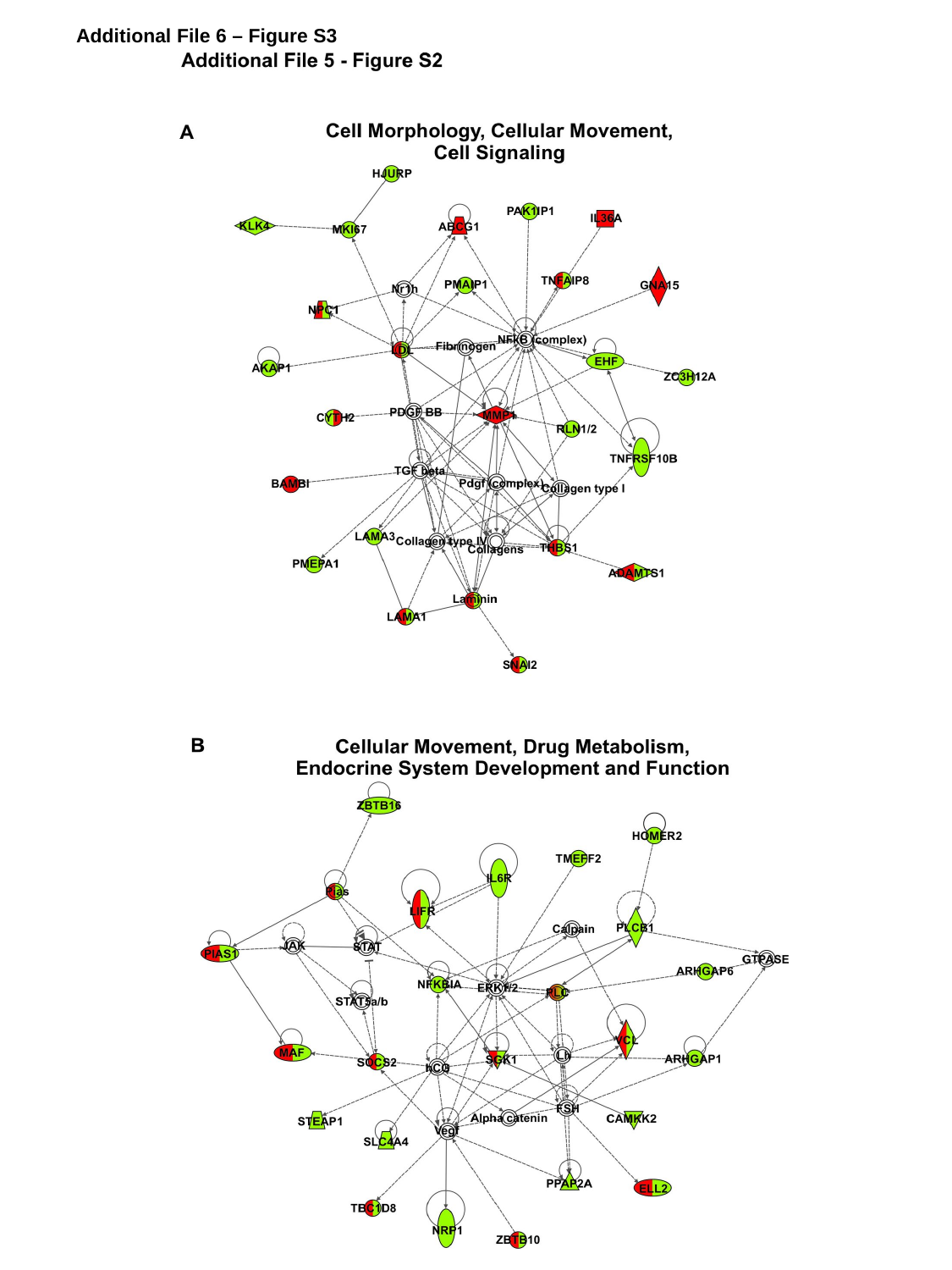

Additional File 6 – Figure S3

Supplement: Additional file 6: Figure S3 — Enriched networks in list of 216 genes with enhanced regulation by the combination. The network tool in Ingenuity Pathway Analysis (IPA) identified the networks of Cell Morphology, Cellular Movement and Cell Signaling (A), as well as Cellular Movement, Drug Metabolism, Endocrine System Development and Function (B), as the two most significantly enriched by the combination treatment when compared to the individual agents (list of 216 genes). Colors indicate the regulation by the combination when compared to both of the individual agents – green is downregulation, red is upregulation and half green half red means that the combination upregulated the gene compared to one treatment, and downregulated compared to the other. The lines in between the genes represent a network connection. (PPTX 18374 kb) [file 12885_2016_2188_MOESM6_ESM.pptx]

## Slide 1
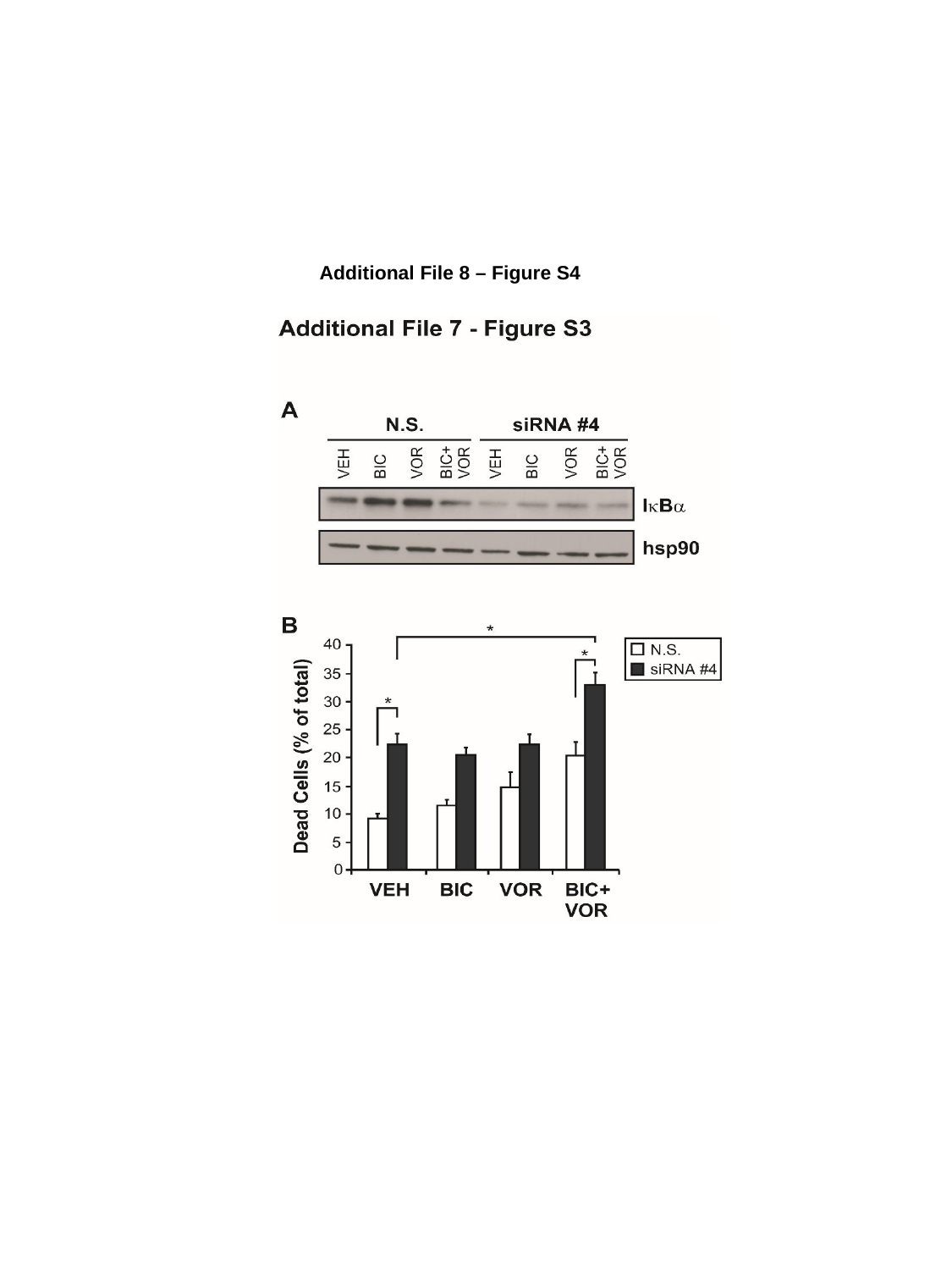

Additional File 8 – Figure S4

Supplement: Additional file 8: Figure S4. — Knockdown of NFKBIA enhances the cell death effect observed with the combination therapy. (A) Western blot analysis of lysates from LNCaP cells transfected with either non-specific [N.S.] or specific [siRNA #1 and siRNA #4] NFKBIA siRNA and then treated with vehicle control [VC], 2.5 μM bicalutamide [BIC], 1 μM vorinostat [VOR], or the combination of 2.5 μM bicalutamide and 1 μM vorinostat [BIC + VOR]. Steady state levels of IκBα at day three of treatment are shown, with hsp90 as a loading control. (B) Cells were counted at three days of treatment, and assessed for viability using trypan blue dye exclusion. Cell death is expressed as a percentage of total cell number. Values indicated are the mean of triplicate wells ± SEM, and are representative of three independent experiments. * = p < 0.05 using t-test. (PPTX 213 kb) [file 12885_2016_2188_MOESM8_ESM.pptx]
