# Supplementary material for: IκBα mediates prostate cancer cell death induced by combinatorial targeting of the androgen receptor
Source: BMC Cancer. 2016 Feb 23;16:141. doi: 10.1186/s12885-016-2188-2 (PMC4785192; doi:10.1186/s12885-016-2188-2)
Supplement: Additional file 2: Table S1. — Primer Sequences. (DOCX 13 kb) [file 12885_2016_2188_MOESM2_ESM.docx]

**Additional File 2 – Table S1: Primer Sequences**

| Primer name | Primer Sequence |
| --- | --- |
| PMEPA1 forward | GTCTGCACGGTCCTTTGCTC |
| PMEPA1 reverse | CGTTGCGCCCTGCAGATCCT |
| PGM2L1 forward | GGG CAG TGG CTC CGC TGG GAT AA |
| PGM2L1 reverse | CTGCCCCCATGGCAGAACGAAGT |
| STEAP1 forward | GAAGAGTGGGTGGCTGAAGCCATAC |
| STEAP1 reverse | ATGCTGGTCTCTCCCGTGTCCTTA |
| KLK2 forward | GGTGGCTGTGTACAGTCATGGAT |
| KLK2 reverse | TGTCTTCAGGCTCAAACAGGTTG |
| KLK3 forward | ACCAGAGGAGTTCTTGACCCCAAA |
| KLK3 reverse | CCCCAGAATCACCCGAGCAG |
| NKX3-1 forward | CTG GCA GAG ACC GAG CCA GAA AG |
| NKX3-1 reverse | AGC GCT TCT GCG GCT GCT TAG |
| IGF1R forward | TTACTTCTGCTCAGATGCTCCAA |
| IGF1R reverse | TCGATGAGCCCCATGAAGTT |
| NFKBIA forward | ATGTGGACGACCGCCACGACA |
| NFKBIA reverse | ATGGCCAAGTGCAGGAACGAGTC |
| C1orf116 forward | AGCCACAACTCCCAGAGGTTT |
| C1orf116 reverse | TCGTCCTTGCTGAGTGATGG |
| TP53INP1 forward | CGTCTGGGTACCTGAACGAGGTG |
| TP53INP1 reverse | GGAGATTAAAGTGCACAGGGTGCTT |
| CDKN1A forward | TGGACCTGGAGACTCTCAGGGTCG |
| CDKN1A reverse | TTAGGGCTTCCTCTTGGAGAAGATC |
| HPRT1 forward | GTTATGGCGACCCGCAG |
| HPRT1 reverse | ACCCTTTCCAAATCCTCAGC |
| RPL19 forward | TGCCAGTGGAAAAATCAGCCA |
| RPL19 reverse | CAAAGCAAATCTCGACACCTTG |
| GUSB forward | CGTCCCACCTAGAATCTGCT |
| GUSB reverse | TTGCTCACAAAGGTCACAGG |
