# Supplementary material for: IκBα mediates prostate cancer cell death induced by combinatorial targeting of the androgen receptor
Source: BMC Cancer. 2016 Feb 23;16:141. doi: 10.1186/s12885-016-2188-2 (PMC4785192; doi:10.1186/s12885-016-2188-2)
Supplement: Additional file 4: Table S2. — List of 216 genes significantly regulated by combination therapy versus each individual agent. (DOCX 28 kb) [file 12885_2016_2188_MOESM4_ESM.docx]

**Additional File 4 – Table S2: List of 216 genes significantly regulated by combination therapy versus each individual agent.**

|  | | **COMBINATION vs VORINOSTAT** | | **COMBINATION vs BICALUTAMIDE** | |
| --- | --- | --- | --- | --- | --- |
| **Gene name** | **Refseq** | **fold change** | **p value** | **fold change** | **p-value** |
| NKX3-1 | NM_006167 | -2.85263 | 4.03917E-15 | -1.71618 | 3.01878E-09 |
| C1orf116 | NM_023938 | -2.29797 | 1.00377E-13 | -1.2955 | 0.000131532 |
| SNAI2 | NM_003068 | -2.26569 | 1.37528E-08 | 2.44675 | 1.53482E-10 |
| KLK2 | NM_001002231 | -2.18714 | 4.32992E-17 | -1.67071 | 3.45062E-13 |
| ZBTB16 | NM_006006 | -2.06001 | 1.80277E-15 | -1.18913 | 0.000464414 |
| NFKBIA | NM_020529 | -1.99927 | 2.89549E-12 | -1.73419 | 7.52679E-11 |
| SLC45A3 | NM_033102 | -1.94909 | 1.62206E-11 | -1.40117 | 1.90529E-06 |
| PMEPA1 | NM_020182 | -1.94777 | 3.04781E-13 | -1.19992 | 0.00105158 |
| THBS1 | NM_003246 | -1.94256 | 1.62206E-11 | 1.19777 | 0.00516974 |
| GREB1 | NM_014668 | -1.93631 | 2.02681E-11 | -1.66296 | 8.09975E-10 |
| KLK3 | NM_001030047 | -1.88542 | 3.03362E-11 | -1.4109 | 9.25534E-07 |
| IGF1R | NM_000875 | -1.81509 | 2.5694E-06 | 1.28255 | 0.010904 |
| FAM105A | NM_019018 | -1.65066 | 6.59152E-08 | 1.61776 | 9.73339E-09 |
| ERRFI1 | NM_018948 | -1.63259 | 1.76756E-09 | 1.15713 | 0.0119312 |
| C19orf48 | NM_199249 | -1.61513 | 1.77213E-06 | -1.42754 | 1.68933E-05 |
| GSTM2 | AK299482 | -1.60376 | 0.00443709 | -1.4744 | 0.00258998 |
| MAF | NM_001031804 | -1.54978 | 8.57574E-06 | 1.21777 | 0.0124486 |
| KCNN2 | NM_021614 | -1.53408 | 8.22921E-09 | -1.24277 | 8.65643E-05 |
| DEFB132 | NM_207469 | -1.52821 | 0.000332638 | -1.55176 | 1.32244E-05 |
| MTMR9 | NM_015458 | -1.51593 | 1.00358E-08 | 1.23868 | 8.53063E-05 |
| ANKRD11 | NM_013275 | -1.51363 | 0.0233201 | -1.246 | 0.00261579 |
| PMAIP1 | NM_021127 | -1.48284 | 7.61257E-06 | -1.38986 | 9.60643E-06 |
| GUCY1A3 | NM_000856 | -1.46278 | 4.88019E-11 | -2.09312 | 6.9488E-19 |
| PMCHL2 | NR_003922 | -1.46033 | 0.000166825 | -1.4456 | 1.63806E-05 |
| LOC440905 | BC144438 | -1.44438 | 0.000518842 | -1.63341 | 3.13904E-07 |
| SLC16A6 | NM_004694 | -1.44186 | 1.19851E-06 | -1.58817 | 6.54705E-10 |
| PAK1IP1 | NM_017906 | -1.43754 | 2.96653E-07 | -1.22247 | 0.000258585 |
| EHF | NM_012153 | -1.43242 | 0.000118337 | -1.16633 | 0.0468649 |
| MAK | NM_005906 | -1.43061 | 5.81896E-05 | 1.24288 | 0.00246284 |
| RHOU | NM_021205 | -1.42853 | 8.54787E-09 | 1.54736 | 7.90368E-12 |
| PRAGMIN | NM_001080826 | -1.4272 | 0.000394532 | 1.18041 | 0.0461671 |
| TMEFF2 | NM_016192 | -1.42413 | 1.59171E-06 | -1.27494 | 4.67987E-05 |
| RBM24 | NM_001143942 | -1.42369 | 0.000182169 | -1.28177 | 0.00116033 |
| POTEF | NM_001099771 | -1.42199 | 0.000017682 | -1.14076 | 0.00815964 |
| CDC42EP3 | NM_006449 | -1.4116 | 0.000619201 | 1.45215 | 1.31468E-05 |
| POTEE | NM_001083538 | -1.4075 | 1.75241E-05 | -1.36046 | 6.6062E-06 |
| STEAP1 | NM_012449 | -1.40644 | 0.0299445 | -1.63868 | 2.97397E-05 |
| PCOTH | NM_001014442 | -1.39672 | 0.000780485 | -1.31468 | 0.000668641 |
| C11orf92 | NM_207429 | -1.38919 | 7.61257E-06 | -1.17762 | 0.00507725 |
| CYP3A5 | NM_000777 | -1.38189 | 0.00813625 | 1.32818 | 0.00241973 |
| ADAM7 | NM_003817 | -1.37649 | 0.000017682 | -1.5865 | 1.08094E-09 |
| NRP1 | NM_003873 | -1.36951 | 6.17256E-06 | -1.7328 | 4.37988E-12 |
| C2orf14 | NR_023391 | -1.36558 | 2.5694E-06 | -1.57738 | 8.2715E-11 |
| KLK15 | NM_017509 | -1.36448 | 3.57777E-06 | -1.132 | 0.0205857 |
| SLC26A2 | NM_000112 | -1.36339 | 0.00498382 | 1.20191 | 0.0353808 |
| TIGD6 | NM_030953 | -1.36243 | 0.00126287 | -1.6648 | 1.91442E-08 |
| FAM65B | NM_014722 | -1.3601 | 0.00103024 | -1.53107 | 3.83991E-07 |
| KCNRG | NM_173605 | -1.35861 | 0.0168558 | -1.38481 | 0.000696497 |
| SLC36A1 | NM_078483 | -1.35667 | 0.000460334 | 1.38383 | 1.21549E-05 |
| POTEH | NM_001136213 | -1.3528 | 7.84713E-05 | -1.13923 | 0.0389797 |
| POTEG | NR_027480 | -1.3517 | 4.23205E-05 | -1.16338 | 0.0276417 |
| GRHL2 | NM_024915 | -1.35168 | 4.83588E-07 | -1.12894 | 0.0088105 |
| ZBTB10 | NM_001105539 | -1.35026 | 4.95954E-05 | 1.34762 | 3.5931E-06 |
| STAG3L4 | NM_022906 | -1.34978 | 0.00770934 | -1.60766 | 7.03512E-07 |
| NSMAF | NM_003580 | -1.34298 | 1.21493E-05 | -1.84715 | 2.18716E-13 |
| ARHGAP6 | NM_013427 | -1.34097 | 0.000234727 | -1.50337 | 5.33969E-08 |
| ELK4 | NM_001973 | -1.33268 | 1.12727E-05 | -1.32879 | 8.63354E-07 |
| CENPN | NM_001100624 | -1.32956 | 0.000248414 | -1.54311 | 9.81429E-09 |
| RASSF3 | NM_178169 | -1.32943 | 0.00697337 | -1.2492 | 0.0060122 |
| PPFIBP1 | NM_003622 | -1.32768 | 1.70814E-05 | 1.11902 | 0.037655 |
| LRRC63 | ENST00000446175 | -1.32554 | 0.00477314 | 1.39475 | 3.73919E-05 |
| KLK4 | NM_004917 | -1.32366 | 1.70814E-05 | -1.1705 | 0.00261079 |
| TNFAIP8 | NM_014350 | -1.32192 | 0.00323789 | 1.33784 | 0.000128873 |
| ZFX | NM_003410 | -1.31983 | 0.0189417 | -2.19773 | 4.09816E-11 |
| IFIT5 | NM_012420 | -1.31619 | 0.0150013 | -1.26417 | 0.00549174 |
| SLC6A8 | NM_005629 | -1.31535 | 0.0168558 | -1.29532 | 0.00239821 |
| LOC388152 | NR_027001 | -1.3133 | 2.55963E-06 | 1.13484 | 0.00555988 |
| LRRFIP2 | NM_006309 | -1.30955 | 0.000332638 | 1.21473 | 0.00137211 |
| TBC1D8 | NM_001102426 | -1.30923 | 0.000229382 | 1.26004 | 0.000131386 |
| FLJ39632 | AK096951 | -1.30909 | 0.0172272 | -1.24091 | 0.0104091 |
| LOC80154 | NR_026811 | -1.30735 | 1.31582E-06 | 1.1601 | 0.000648975 |
| ZNF649 | NM_023074 | -1.30631 | 0.00477314 | -1.57161 | 9.11615E-08 |
| ELL2 | NM_012081 | -1.30432 | 1.70814E-05 | 1.22982 | 5.44546E-05 |
| SLC41A1 | NM_173854 | -1.30335 | 0.000303451 | 1.32864 | 6.97019E-06 |
| PHACTR2 | NM_014721 | -1.30058 | 0.00231945 | -1.2224 | 0.00311677 |
| HOMER2 | NM_199330 | -1.30011 | 0.00753275 | -1.18537 | 0.0257886 |
| C11orf82 | NM_145018 | -1.29855 | 0.000166825 | 1.17739 | 0.00347285 |
| TRIM13 | NM_213590 | -1.29689 | 0.011402 | -1.37265 | 0.00007642 |
| PTGER4 | NM_000958 | -1.2968 | 0.00391548 | 1.31152 | 0.000154434 |
| DNAJB14 | NM_001031723 | -1.29486 | 0.0221605 | 1.14474 | 0.0311174 |
| DTX3L | NM_138287 | -1.29121 | 0.0106551 | -2.74342 | 5.21202E-15 |
| CENPL | NM_001127181 | -1.2898 | 0.00512119 | -1.16591 | 0.0324195 |
| KIAA1731 | NM_033395 | -1.28888 | 4.57265E-05 | -1.18555 | 0.000756414 |
| FGD4 | NM_139241 | -1.28885 | 0.00559844 | 1.26182 | 0.0010652 |
| KIAA0040 | NM_014656 | -1.28504 | 0.0440293 | 1.42889 | 7.60315E-05 |
| NAT1 | NM_001160170 | -1.28428 | 0.00559844 | 1.23406 | 0.00257767 |
| SNRPD1 | NM_006938 | -1.28364 | 0.0495629 | -1.3947 | 0.000231525 |
| NTNG1 | NM_001113226 | -1.28259 | 0.0136633 | -1.49772 | 1.21165E-06 |
| IL6R | NM_000565 | -1.28216 | 0.000802121 | -1.73353 | 2.10954E-11 |
| PDE9A | NM_002606 | -1.28092 | 0.000189173 | -1.15636 | 0.0066834 |
| SOCS2 | NM_003877 | -1.28048 | 0.00165702 | 1.19372 | 0.00450061 |
| NPC1 | NM_000271 | -1.28038 | 1.94242E-06 | 1.4947 | 5.45659E-12 |
| C1orf21 | NM_030806 | -1.28031 | 0.000676027 | -1.12758 | 0.0454704 |
| ABHD2 | NM_007011 | -1.27833 | 2.51813E-09 | -1.15052 | 7.85656E-06 |
| SGEF | NM_015595 | -1.27724 | 0.0242508 | -1.31422 | 0.00063356 |
| ORC5L | NM_002553 | -1.27649 | 0.00010039 | 1.18115 | 0.00105469 |
| CAMKK2 | NM_006549 | -1.27503 | 0.00218893 | -1.22391 | 0.00128355 |
| MICAL1 | NM_022765 | -1.27454 | 0.0292598 | 1.32794 | 0.000475872 |
| SPSB1 | NM_025106 | -1.27369 | 0.00760768 | 1.33716 | 5.32521E-05 |
| PDLIM5 | NM_006457 | -1.27335 | 0.000470672 | 1.265 | 4.70538E-05 |
| STK17B | NM_004226 | -1.27082 | 0.0106963 | 1.5394 | 1.22363E-07 |
| POLE2 | NM_002692 | -1.2699 | 0.0088152 | -1.26746 | 0.000732675 |
| ARHGAP28 | NM_001010000 | -1.2693 | 0.0114981 | 1.23976 | 0.00259073 |
| SYNJ1 | NM_003895 | -1.26753 | 0.0193701 | 1.23436 | 0.00495445 |
| LRCH1 | NM_015116 | -1.26055 | 0.0361916 | 1.49526 | 2.4626E-06 |
| FBXO38 | NM_205836 | -1.25961 | 0.000518842 | 1.22145 | 0.000241306 |
| CYB5A | NM_148923 | -1.25737 | 0.0203522 | -1.17528 | 0.028904 |
| SNORD82 | NR_004398 | -1.25229 | 0.0239256 | -1.62732 | 1.8431E-08 |
| SNX24 | NM_014035 | -1.25128 | 0.0436037 | 1.24039 | 0.00582121 |
| FAM13A | NM_014883 | -1.25086 | 0.00468399 | 1.86376 | 2.87891E-12 |
| CDYL2 | NM_152342 | -1.25074 | 0.0121503 | -1.18028 | 0.0143575 |
| PCTP | NM_001102402 | -1.25073 | 0.0434289 | 1.27471 | 0.00184999 |
| RLN1 | NM_006911 | -1.24954 | 0.0038226 | -1.71038 | 4.70142E-11 |
| KIF20A | NM_005733 | -1.24773 | 0.0136247 | -1.36293 | 1.35973E-05 |
| PPM1K | NM_152542 | -1.246 | 0.0141688 | 1.38777 | 5.41003E-06 |
| EFNA5 | NM_001962 | -1.24454 | 0.0256367 | -1.23061 | 0.00356593 |
| LOC388692 | NR_027002 | -1.24437 | 0.046137 | -1.22108 | 0.00952941 |
| SGK1 | NM_001143676 | -1.2443 | 0.00226988 | 1.33403 | 0.000002526 |
| HMGCR | NM_000859 | -1.24328 | 0.00679091 | 1.36278 | 3.41534E-06 |
| LIFR | NM_002310 | -1.24308 | 0.020547 | 1.84466 | 5.8966E-11 |
| LAMA3 | NM_198129 | -1.24125 | 1.75703E-06 | -1.16703 | 2.91644E-05 |
| PPAP2A | NM_003711 | -1.23907 | 0.00403973 | -1.27339 | 5.67999E-05 |
| WAC | NR_024557 | -1.23689 | 0.0487296 | -1.261 | 0.00211193 |
| TAF5L | NM_014409 | -1.23512 | 0.00565963 | -1.32068 | 9.23375E-06 |
| ACSL3 | NM_004457 | -1.23374 | 1.28578E-05 | 1.20724 | 5.18208E-06 |
| DEGS1 | NM_003676 | -1.23296 | 0.0179251 | -1.14515 | 0.0444441 |
| PLCB1 | NM_182734 | -1.23276 | 0.0366559 | -1.17957 | 0.0218053 |
| CEP120 | NM_153223 | -1.23224 | 0.000454345 | 1.16282 | 0.00168347 |
| FNBP1L | NM_001024948 | -1.23211 | 0.0130111 | 1.32246 | 2.47439E-05 |
| TNFRSF10B | NM_003842 | -1.22932 | 0.00217589 | -1.146 | 0.0105049 |
| TRIM49 | NM_020358 | -1.22902 | 0.0171721 | 1.14227 | 0.0296197 |
| STEAP2 | NM_152999 | -1.22745 | 0.00441367 | -1.48198 | 6.64615E-09 |
| DPY30 | NM_032574 | -1.2245 | 0.0160576 | -1.14123 | 0.0395834 |
| UBE2D1 | NM_003338 | -1.22019 | 0.0382366 | 1.37082 | 1.25117E-05 |
| ZNF608 | NM_020747 | -1.21987 | 0.0307706 | 1.37637 | 6.45117E-06 |
| SLC38A2 | NM_018976 | -1.21979 | 0.000619201 | -1.21754 | 4.74618E-05 |
| UNQ9368 | NR_003542 | -1.21858 | 0.0283178 | 1.15068 | 0.034808 |
| C16orf53 | NM_024516 | -1.21702 | 0.0179251 | -1.87725 | 2.38198E-12 |
| FAM111B | NM_198947 | -1.21678 | 0.0026419 | 1.21201 | 0.000235082 |
| LOC285550 | NM_001145191 | -1.21602 | 0.0475263 | -1.42379 | 2.47409E-06 |
| CHD7 | NM_017780 | -1.21437 | 0.00474847 | 1.6144 | 4.29346E-11 |
| ZC3H12A | NM_025079 | -1.21272 | 0.0193701 | -1.49045 | 2.762E-08 |
| CENPO | NM_024322 | -1.21256 | 0.00678114 | -1.29311 | 9.5216E-06 |
| SETMAR | NM_006515 | -1.21104 | 0.00480239 | -1.82389 | 2.18716E-13 |
| ARHGAP1 | NM_004308 | -1.20884 | 0.0186786 | -1.45814 | 5.54669E-08 |
| JAG1 | NM_000214 | -1.2087 | 0.0088152 | 1.2731 | 2.94141E-05 |
| MTAP | NM_002451 | -1.20853 | 0.0104781 | -1.18678 | 0.00229679 |
| ADAMTS1 | NM_006988 | -1.20813 | 0.000158831 | 1.19496 | 2.50011E-05 |
| RAD51AP1 | NM_001130862 | -1.20773 | 0.01297 | -1.13886 | 0.0247796 |
| SLC4A4 | NM_001098484 | -1.20713 | 0.0121503 | -2.28298 | 7.3824E-16 |
| CLU | NM_001831 | -1.20606 | 0.00252481 | 1.41409 | 5.63014E-09 |
| PIAS1 | NM_016166 | -1.20581 | 0.0147346 | 1.33082 | 3.84152E-06 |
| TRPM8 | NM_024080 | -1.2048 | 0.03315 | -1.1684 | 0.0133436 |
| SLC25A37 | NM_016612 | -1.20433 | 0.00152472 | -1.23799 | 1.44455E-05 |
| DNAH5 | NM_001369 | -1.2031 | 0.0118843 | -1.52086 | 1.65706E-09 |
| MAP9 | NM_001039580 | -1.198 | 0.000260986 | 1.26344 | 2.35426E-07 |
| ABCC4 | NM_005845 | -1.1976 | 0.0193701 | -1.16294 | 0.00819867 |
| ASAP1 | NM_018482 | -1.19624 | 0.0216666 | -1.47661 | 1.34963E-08 |
| HEATR6 | NM_022070 | -1.19442 | 0.0452897 | -1.57445 | 3.4437E-09 |
| POLK | NM_016218 | -1.19437 | 0.0452897 | 1.27388 | 0.000144607 |
| HUS1 | NM_004507 | -1.19315 | 0.00745052 | -1.58995 | 2.51043E-11 |
| ZFHX3 | NM_006885 | -1.19175 | 0.034665 | -1.86273 | 1.31423E-12 |
| C2orf3 | NM_003203 | -1.19098 | 0.0372785 | -1.15922 | 0.0136335 |
| THRB | NM_001128176 | -1.18919 | 0.0381349 | -1.53812 | 3.73598E-09 |
| FOXA1 | NM_004496 | -1.18912 | 0.00202986 | -1.66655 | 1.78559E-13 |
| TEX2 | NM_018469 | -1.18457 | 0.0172272 | 1.18506 | 0.00135089 |
| PNMA1 | NM_006029 | -1.18296 | 0.0129595 | 1.32546 | 6.18498E-07 |
| VCL | NM_014000 | -1.18266 | 0.0484857 | 1.36669 | 1.55222E-06 |
| MKI67 | NM_002417 | -1.18264 | 0.0238298 | -1.30865 | 4.06673E-06 |
| AKAP1 | NM_003488 | -1.17966 | 9.43564E-05 | -1.15191 | 6.08622E-05 |
| LIMCH1 | NM_014988 | -1.17844 | 0.0079617 | -1.28749 | 1.19732E-06 |
| HJURP | NM_018410 | -1.17647 | 0.0484857 | -1.26786 | 6.53543E-05 |
| FASN | NM_004104 | -1.17567 | 0.0374548 | -1.15132 | 0.011091 |
| TPT1 | NM_003295 | -1.17344 | 0.0026419 | -1.28656 | 1.37452E-07 |
| BDP1 | NM_018429 | -1.17082 | 0.00247934 | -1.21714 | 7.15654E-06 |
| PHF8 | NM_015107 | -1.16806 | 0.024751 | -1.1125 | 0.0389841 |
| USP46 | NM_022832 | -1.16762 | 0.0345012 | -1.27752 | 1.03017E-05 |
| LAMA1 | NM_005559 | -1.16542 | 0.00768003 | 1.20896 | 0.000035093 |
| RAB6C | NM_032144 | -1.16276 | 0.0265154 | -1.33007 | 2.87615E-07 |
| KIAA1244 | NM_020340 | -1.13492 | 0.00165702 | -1.35123 | 1.46722E-11 |
| PRUNE2 | NM_015225 | -1.13403 | 0.0307473 | -1.4197 | 1.94907E-10 |
| UAP1 | NM_003115 | -1.12791 | 0.0172347 | 1.23959 | 3.5231E-07 |
| HEBP2 | NM_014320 | -1.11861 | 0.0256562 | -1.11445 | 0.00299574 |
| APPBP2 | NM_006380 | -1.11813 | 0.0374571 | 1.15617 | 0.000191647 |
| SORD | NM_003104 | -1.10795 | 0.00537706 | -1.10625 | 0.000459683 |
| TUBB | NM_178014 | -1.07757 | 0.0282495 | -1.13917 | 1.36199E-06 |
| GUSBL1 | BC171739 | 1.11327 | 0.0171929 | -1.16655 | 3.57553E-06 |
| SMA5 | AK289851 | 1.12009 | 0.00134603 | -1.14772 | 5.01879E-06 |
| GUSBL2 | NR_003660 | 1.13571 | 0.00120797 | -1.13162 | 0.000117385 |
| MMP1 | NM_002421 | 1.15926 | 0.0304391 | 1.10598 | 0.0462673 |
| TRIM42 | NM_152616 | 1.16259 | 0.0361803 | 1.14018 | 0.0105706 |
| CACNA2D1 | NM_000722 | 1.16731 | 0.0215678 | 1.1314 | 0.0129334 |
| PLEKHH2 | NM_172069 | 1.17777 | 0.045188 | 1.26206 | 0.000080112 |
| TFAP2B | NM_003221 | 1.18459 | 0.0287645 | 1.14901 | 0.0134207 |
| EPB41L4A | NM_022140 | 1.18684 | 0.0223049 | 1.17029 | 0.00420387 |
| CYTH2 | NM_017457 | 1.18754 | 0.0131972 | -1.27475 | 0.00024265 |
| CD8B | NM_172099 | 1.19557 | 0.039108 | 1.13767 | 0.0404713 |
| FAM167A | NM_053279 | 1.20637 | 0.00760768 | 1.28625 | 0.000010588 |
| SIDT1 | NM_017699 | 1.21484 | 0.03315 | 1.38019 | 4.50727E-06 |
| C12orf60 | NM_175874 | 1.22378 | 0.0484857 | 1.2154 | 0.00643523 |
| GNA15 | NM_002068 | 1.22852 | 0.0383984 | 1.19226 | 0.0131736 |
| TP53INP1 | NM_033285 | 1.23274 | 0.000706435 | 1.18673 | 0.000622981 |
| FYN | NM_002037 | 1.2355 | 0.00273241 | 2.5358 | 2.08151E-17 |
| SYTL2 | NM_206927 | 1.2466 | 0.000626777 | 1.15691 | 0.0048956 |
| AVIL | NM_006576 | 1.25662 | 0.0422237 | 1.22889 | 0.00945533 |
| IL1F6 | NM_014440 | 1.26047 | 0.00568403 | 1.15234 | 0.0313232 |
| LOC143188 | NR_015409 | 1.26395 | 0.0452897 | 1.23658 | 0.0098958 |
| LIPH | NM_139248 | 1.27535 | 0.0374548 | 2.08284 | 5.9782E-11 |
| GSDMB | NM_001042471 | 1.27842 | 0.00477314 | 2.77277 | 3.17093E-16 |
| KIAA1324 | NM_020775 | 1.30532 | 0.00692554 | 1.41758 | 0.000013169 |
| BAMBI | NM_012342 | 1.31377 | 0.00882871 | 1.31373 | 0.000678913 |
| ABCG1 | NM_207627 | 1.316 | 0.00403973 | 1.29473 | 0.000579727 |
| SLC7A11 | NM_014331 | 1.32953 | 9.86063E-06 | -1.42286 | 1.14536E-08 |
| HBEGF | NM_001945 | 1.33382 | 0.0141688 | 1.62228 | 7.18267E-07 |
| CHAC1 | NM_024111 | 1.33858 | 0.0374548 | -1.81066 | 1.63379E-07 |
| DDIT3 | NM_004083 | 1.34439 | 0.00669707 | -1.33003 | 0.000724264 |
