# Supplementary material for: IκBα mediates prostate cancer cell death induced by combinatorial targeting of the androgen receptor
Source: BMC Cancer. 2016 Feb 23;16:141. doi: 10.1186/s12885-016-2188-2 (PMC4785192; doi:10.1186/s12885-016-2188-2)
Supplement: Additional file 5: Table S3. — Comparison of pathway analyses for bicalutamide, vorinostat, or the combination versus vehicle control. KEGG pathways. (DOCX 24 kb) [file 12885_2016_2188_MOESM5_ESM.docx]

**Additional File 5 – Table S3: Comparison of pathway analyses for bicalutamide, vorinostat, or the combination versus vehicle control.**

**KEGG pathways**

| **Bicalutamide vs Vehicle Control** | | **Vorinostat vs Vehicle Control** | | **Combination vs Vehicle Control** | |
| --- | --- | --- | --- | --- | --- |
| *KEGG Pathway Term* | *p-value* | *KEGG Pathway Term* | *p-value* | *KEGG Pathway Term* | *p-value* |
| hsa05222: Small cell lung cancer | 0.0022 | hsa03022: Basal transcription factors | 8.45E-06 | hsa00562: Inositol phosphate metabolism | 7.25E-07 |
| hsa00512: O-Glycan biosynthesis | 0.0069 | hsa04210: Apoptosis | 5.50E-05 | hsa04210: Apoptosis | 1.83E-06 |
| hsa05200: Pathways in cancer | 0.0075 | hsa04520: Adherens junction | 7.48E-05 | hsa04070: Phosphatidylinositol signaling system | 1.23E-05 |
| hsa05215: Prostate cancer | 0.0104 | hsa04115: p53 signaling pathway | 9.47E-05 | hsa04130: SNARE interactions in vesicular transport | 1.66E-04 |
| hsa04920: Adipocytokine signaling pathway | 0.0194 | hsa00562: Inositol phosphate metabolism | 4.00E-04 | hsa04110: Cell cycle | 1.78E-04 |
| hsa00500: Starch and sucrose metabolism | 0.0253 | hsa05200: Pathways in cancer | 5.33E-04 | hsa00310: Lysine degradation | 2.97E-04 |
| hsa04960: Aldosterone-regulated sodium reabsorption | 0.0253 | hsa04070: Phosphatidylinositol signaling system | 7.63E-04 | hsa05223: Non-small cell lung cancer | 7.99E-04 |
| hsa00053: Ascorbate and aldarate metabolism | 0.0259 | hsa04110: Cell cycle | 0.0011 | hsa04144: Endocytosis | 0.0012 |
| hsa00040: Pentose and glucuronate interconversions | 0.0305 | hsa04130: SNARE interactions in vesicular transport | 0.0021 | hsa03022: Basal transcription factors | 0.0014 |
| hsa04730: Long-term depression | 0.0633 | hsa05210: Colorectal cancer | 0.0027 | hsa04520: Adherens junction | 0.0017 |
| hsa04012: ErbB signaling pathway | 0.0660 | hsa05120: Epithelial cell signaling in Helicobacter pylori infection | 0.0031 | hsa00100: Steroid biosynthesis | 0.0023 |
| hsa04360: Axon guidance | 0.0833 | hsa05213: Endometrial cancer | 0.0032 | hsa04115: p53 signaling pathway | 0.0024 |
| hsa05221: Acute myeloid leukemia | 0.0840 | hsa05212: Pancreatic cancer | 0.0044 | hsa04914: Progesterone-mediated oocyte maturation | 0.0026 |
| hsa04662: B cell receptor signaling pathway | 0.0872 | hsa05215: Prostate cancer | 0.0046 | hsa03410: Base excision repair | 0.0026 |
| hsa00983: Drug metabolism | 0.0927 | hsa04120: Ubiquitin mediated proteolysis | 0.0055 | hsa04710: Circadian rhythm | 0.0030 |
| hsa05223: Non-small cell lung cancer | 0.0059 | hsa05211: Renal cell carcinoma | 0.0031 |  |  |
| hsa00310: Lysine degradation | 0.0074 | hsa04910: Insulin signaling pathway | 0.0032 |  |  |
| hsa00280: Valine, leucine and isoleucine degradation | 0.0074 | hsa05215: Prostate cancer | 0.0034 |  |  |
| hsa04330: Notch signaling pathway | 0.0082 | hsa04666: Fc gamma R-mediated phagocytosis | 0.0037 |  |  |
| hsa05211: Renal cell carcinoma | 0.0083 | hsa05200: Pathways in cancer | 0.0062 |  |  |
| hsa05220: Chronic myeloid leukemia | 0.0091 | hsa05212: Pancreatic cancer | 0.0067 |  |  |
| hsa00100: Steroid biosynthesis | 0.0106 | hsa05222: Small cell lung cancer | 0.0077 |  |  |
| hsa05222: Small cell lung cancer | 0.0181 | hsa04722: Neurotrophin signaling pathway | 0.0149 |  |  |
| hsa04142: Lysosome | 0.0200 | hsa04120: Ubiquitin mediated proteolysis | 0.0172 |  |  |
| hsa03040: Spliceosome | 0.0201 | hsa00450: Selenoamino acid metabolism | 0.0182 |  |  |
| hsa00450: Selenoamino acid metabolism | 0.0209 | hsa05120: Epithelial cell signaling in Helicobacter pylori infection | 0.0192 |  |  |
| hsa05216:Thyroid cancer | 0.0239 | hsa05214: Glioma | 0.0228 |  |  |
| hsa00860: Porphyrin and chlorophyll metabolism | 0.0265 | hsa03040: Spliceosome | 0.0237 |  |  |
| hsa04012: ErbB signaling pathway | 0.0313 | hsa00510: N-Glycan biosynthesis | 0.0238 |  |  |
| hsa04914: Progesterone-mediated oocyte maturation | 0.0369 | hsa05220: Chronic myeloid leukemia | 0.0242 |  |  |
| hsa00600: Sphingolipid metabolism | 0.0404 | hsa04012: ErbB signaling pathway | 0.0248 |  |  |
| hsa03020: RNA polymerase | 0.0410 | hsa04010: MAPK signaling pathway | 0.0268 |  |  |
| hsa04910: Insulin signaling pathway | 0.0461 | hsa04960: Aldosterone-regulated sodium reabsorption | 0.0281 |  |  |
| hsa05110: Vibrio cholerae infection | 0.0474 | hsa00920: Sulfur metabolism | 0.0313 |  |  |
|  |  | hsa05219: Bladder cancer | 0.0362 |  |  |
|  |  | hsa04114: Oocyte meiosis | 0.0391 |  |  |
|  |  | hsa05110: Vibrio cholerae infection | 0.0401 |  |  |
|  |  | hsa04150: mTOR signaling pathway | 0.0486 |  |  |
|  |  | hsa05213: Endometrial cancer | 0.0486 |  |  |
|  |  | hsa05130: Pathogenic Escherichia coli infection | 0.0491 |  |  |

**Gene Set Enrichment Analysis (GSEA) pathways**

| **Bicalutamide vs Vehicle Control** | **Vorinostat vs Vehicle Control** | | **Combination vs Vehicle Control** | |
| --- | --- | --- | --- | --- |
| GSEA DOWN | GSEA UP | | GSEA UP | GSEA DOWN |
| HSA04730: LONG TERM DEPRESSION  *(p-value 0)* | HSA01030: GLYCAN STRUCTURES BIOSYNTHESIS 1  *(p-value 0)* | HSA01032: GLYCAN STRUCTURES DEGRADATION  *(p-value 0.031621)* | HSA01030: GLYCAN STRUCTURES BIOSYNTHESIS 1  *(p-value 0)* | HSA00150: ANDROGEN AND ESTROGEN METABOLISM  *(p-value 0.003861)* |
| HSA04930: TYPE II DIABETES MELLITUS  *(p-value 0.00198)* | HSA04530: TIGHT JUNCTION  *(p-value 0)* | HSA04012: ERBB SIGNALING PATHWAY  *(p-value 0.034908)* | HSA00534: HEPARAN SULFATE BIOSYNTHESIS  *(p-value 0.003846)* |  |
| HSA04630: JAK STAT SIGNALING PATHWAY  *(p-value 0.00206)* | HSA05120: EPITHELIAL CELL SIGNALING IN HELICOBACTER PYLORI INFECTION  *(p value 0.005906)* | HSA05110: CHOLERA INFECTION  *(p-value 0.0355)* | HSA00330: ARGININE AND PROLINE METABOLISM  *(p-value 0.007937)* |  |
| HSA00641: 3 CHLOROACRYLIC ACID DEGRADATION  *(p-value 0.0125)* | HSA04360: AXON GUIDANCE  *(p-value 0.006036)* | HSA00533: KERATAN SULFATE BIOSYNTHESIS  *(p-value 0.04208)* | HSA05120: EPITHELIAL CELL SIGNALING IN HELICOBACTER PYLORI INFECTION  *(p-value 0.01004)* |  |
| HSA04520: ADHERENS JUNCTION  *(p-value 0.018)* | HSA04660: T CELL RECEPTOR SIGNALING PATHWAY  *(p-value 0.009747)* | HSA00720: REDUCTIVE CARBOXYLATE CYCLE  *(p-value 0.04365)* | HSA04530: TIGHT JUNCTION  *(p-value 0.01417)* |  |
| HSA00980: METABOLISM OF XENOBIOTICS BY CYTOCHROME P450  *(p-value 0.02692)* | HSA05130: PATHOGENIC ESCHERICHIA COLI INFECTION EHEC  *(p-value 0.009921)* | HSA04910: INSULIN SIGNALING PATHWAY  *(p-value 0.04536)* | HSA04360: AXON GUIDANCE  *(p-value 0.020243)* |  |
| HSA00760: NICOTINATE AND NICOTINAMIDE METABOLISM  *(p-value 0.03)* | HSA05131: PATHOGENIC ESCHERICHIA COLI INFECTION EPEC  *(p-value 0.009921)* | HSA00534: HEPARAN SULFATE BIOSYNTHESIS  *(p-value 0.04573)* | HSA04670: LEUKOCYTE TRANSENDOTHELIAL MIGRATION *(p-value 0.02439)* |  |
| HSA05215: PROSTATE CANCER  *(p-value 0.03107)* | HSA00251: GLUTAMATE METABOLISM  *(p-value 0.01)* | HSA00511: N GLYCAN DEGRADATION  *(p-value 0.04609)* | HSA05130: PATHOGENIC ESCHERICHIA COLI INFECTION EHEC  *(p-value 0.02729)* |  |
| HSA05218: MELANOMA  *(p-value 0.03306)* | HSA00220: UREA CYCLE AND METABOLISM OF AMINO GROUPS  *(p-value 0.010267)* | HSA04070: PHOSPHATIDYLINOSITOL SIGNALING SYSTEM  *(p-value 0.04619)* | HSA05131: PATHOGENIC ESCHERICHIA COLI INFECTION EPEC *(p-value 0.02729)* |  |
| HSA05210: COLORECTAL CANCER  *(p-value 0.03861)* | HSA00330: ARGININE AND PROLINE METABOLISM  *(p-value 0.024291)* | HSA00510: N GLYCAN BIOSYNTHESIS  *(p-value 0.04647)* | HSA04660: T CELL RECEPTOR SIGNALING PATHWAY  *(p-value 0.033333)* |  |
|  | HSA05010: ALZHEIMERS DISEASE  *(p-value 0.026)* |  | HSA04070: PHOSPHATIDYLINOSITOL SIGNALING SYSTEM  *(p-value 0.037657)* |  |
|  | HSA00600: SPHINGOLIPID METABOLISM  *(p-value 0.031068)* |  | HSA05110: CHOLERA INFECTION  *(p-value 0.038462)* |  |
|  |  |  | HSA00720: REDUCTIVE CARBOXYLATE CYCLE  *(p-value 0.044898)* |  |

**Ingenuity Pathway Analysis (IPA) pathways**

| **Bicalutamide vs Vehicle Control** | | **Vorinostat vs Vehicle Control** | | **Combination vs Vehicle Control** | |
| --- | --- | --- | --- | --- | --- |
| *Ingenuity canonical pathway* | *(p-value)* | *Ingenuity canonical pathway* | *(p-value)* | *Ingenuity canonical pathway* | *(p-value)* |
| TNFR2 Signaling | 1.45E-03 | Molecular Mechanisms of Cancer | 7.41E-08 | Inositol Phosphate Metabolism | 2.75E-08 |
| Type II Diabetes Mellitus Signaling | 1.70E-03 | Hereditary Breast Cancer Signaling | 2.82E-05 | p53 Signaling | 3.39E-08 |
| Nicotinate and Nicotinamide Metabolism | 4.79E-03 | Prostate Cancer Signaling | 7.59E-05 | Hereditary Breast Cancer Signaling | 8.91E-06 |
| MIF Regulation of Innate Immunity | 8.13E-03 | Cyclins and Cell Cycle Regulation | 9.12E-05 | Molecular Mechanisms of Cancer | 1.15E-05 |
| O-Glycan Biosynthesis | 1.45E-02 | RANK Signaling in Osteoclasts | 1.58E-04 | Death Receptor Signaling | 1.70E-05 |
| Death Receptor Signaling | 1.48E-02 | Regulation of eIF4 and p70S6K Signaling | 1.70E-04 | ATM Signaling | 4.47E-05 |
| Neuregulin Signaling | 1.58E-02 | p53 Signaling | 2.95E-04 | RANK Signaling in Osteoclasts | 5.13E-05 |
| Induction of Apoptosis by HIV1 | 1.78E-02 | Inositol Phosphate Metabolism | 3.09E-04 | Germ Cell-Sertoli Cell Junction Signaling | 8.51E-05 |
| TNFR1 Signaling | 2.00E-02 | Estrogen Receptor Signaling | 3.63E-04 | CD27 Signaling in Lymphocytes | 8.51E-05 |
| mTOR Signaling | 2.63E-02 | Small Cell Lung Cancer Signaling | 3.72E-04 | Role of CHK Proteins in Cell Cycle Checkpoint Control | 8.91E-05 |
| p53 Signaling | 2.69E-02 | ATM Signaling | 4.68E-04 | Role of BRCA1 in DNA Damage Response | 1.15E-04 |
| April Mediated Signaling | 2.88E-02 | Assembly of RNA Polymerase II Complex | 5.50E-04 | Prostate Cancer Signaling | 1.48E-04 |
| B Cell Activating Factor Signaling | 3.47E-02 | Non-Small Cell Lung Cancer Signaling | 6.31E-04 | TWEAK Signaling | 1.86E-04 |
| Growth Hormone Signaling | 3.55E-02 | HGF Signaling | 7.76E-04 | Small Cell Lung Cancer Signaling | 3.02E-04 |
| Glycine, Serine and Threonine Metabolism | 3.55E-02 | TWEAK Signaling | 7.76E-04 | Induction of Apoptosis by HIV1 | 3.47E-04 |
| RAR Activation | 3.72E-02 | Chronic Myeloid Leukemia Signaling | 9.77E-04 | Non-Small Cell Lung Cancer Signaling | 5.13E-04 |
| 4-1BB Signaling in T Lymphocytes | 4.17E-02 | Induction of Apoptosis by HIV1 | 1.05E-03 | Estrogen Receptor Signaling | 5.25E-04 |
| IL-12 Signaling and Production in Macrophages | 4.17E-02 | VEGF Signaling | 1.23E-03 | Insulin Receptor Signaling | 5.75E-04 |
| Inositol Phosphate Metabolism | 4.57E-02 | Death Receptor Signaling | 1.26E-03 | SAPK/JNK Signaling | 7.08E-04 |
| Pentose and Glucuronate Interconversions | 4.68E-02 | Germ Cell-Sertoli Cell Junction Signaling | 1.29E-03 | Aldosterone Signaling in Epithelial Cells | 7.59E-04 |
| Production of Nitric Oxide and Reactive Oxygen Species in Macrophages | 4.90E-02 | Polyamine Regulation in Colon Cancer | 1.51E-03 | Lymphotoxin β Receptor Signaling | 8.71E-04 |
| LPS-stimulated MAPK Signaling | 4.90E-02 | Pancreatic Adenocarcinoma Signaling | 1.82E-03 | Apoptosis Signaling | 1.00E-03 |
| Prolactin Signaling | 4.90E-02 | CD27 Signaling in Lymphocytes | 2.14E-03 | Mitotic Roles of Polo-Like Kinase | 1.05E-03 |
|  |  | PI3K/AKT Signaling | 2.29E-03 | HGF Signaling | 1.23E-03 |
|  |  | Renal Cell Carcinoma Signaling | 2.29E-03 | Glioma Signaling | 1.35E-03 |
|  |  | Role of BRCA1 in DNA Damage Response | 2.51E-03 | 14-3-3-mediated Signaling | 2.14E-03 |
|  |  | CD40 Signaling | 2.82E-03 | GNRH Signaling | 2.14E-03 |
|  |  | Mitotic Roles of Polo-Like Kinase | 2.95E-03 | NRF2-mediated Oxidative Stress Response | 2.19E-03 |
|  |  | TNFR1 Signaling | 3.02E-03 | B Cell Receptor Signaling | 2.34E-03 |
|  |  | Protein Ubiquitination Pathway | 3.39E-03 | TNFR1 Signaling | 2.63E-03 |
|  |  | Myc Mediated Apoptosis Signaling | 4.07E-03 | Pancreatic Adenocarcinoma Signaling | 2.75E-03 |
|  |  | Apoptosis Signaling | 4.90E-03 | Role of PKR in Interferon Induction and Antiviral Response | 3.09E-03 |
|  |  | Aryl Hydrocarbon Receptor Signaling | 6.31E-03 | Chronic Myeloid Leukemia Signaling | 3.16E-03 |
|  |  | ERK/MAPK Signaling | 6.46E-03 | TR/RXR Activation | 3.16E-03 |
|  |  | SAPK/JNK Signaling | 6.61E-03 | Breast Cancer Regulation by Stathmin1 | 3.39E-03 |
|  |  | Endometrial Cancer Signaling | 6.92E-03 | AMPK Signaling | 3.39E-03 |
|  |  | Prolactin Signaling | 6.92E-03 | PPARα/RXRα Activation | 3.39E-03 |
|  |  | Type I Diabetes Mellitus Signaling | 8.32E-03 | Regulation of eIF4 and p70S6K Signaling | 4.07E-03 |
|  |  | Ceramide Signaling | 9.33E-03 | Role of NFAT in Cardiac Hypertrophy | 4.07E-03 |
|  |  | Cell Cycle: G2/M DNA Damage Checkpoint Regulation | 0.010233 | Renal Cell Carcinoma Signaling | 4.07E-03 |
|  |  | Breast Cancer Regulation by Stathmin1 | 0.010471 | FAK Signaling | 4.07E-03 |
|  |  | Role of CHK Proteins in Cell Cycle Checkpoint Control | 0.010471 | Cyclins and Cell Cycle Regulation | 4.07E-03 |
|  |  | IL-6 Signaling | 0.010715 | Granzyme B Signaling | 4.27E-03 |
|  |  | Erythropoietin Signaling | 0.01122 | p70S6K Signaling | 4.27E-03 |
|  |  | Assembly of RNA Polymerase III Complex | 0.011482 | ERK/MAPK Signaling | 5.01E-03 |
|  |  | Synthesis and Degradation of Ketone Bodies | 0.011482 | CD40 Signaling | 5.25E-03 |
|  |  | Pyrimidine Metabolism | 0.012303 | LPS-stimulated MAPK Signaling | 5.75E-03 |
|  |  | Cell Cycle: G1/S Checkpoint Regulation | 0.012303 | Prolactin Signaling | 5.75E-03 |
|  |  | Antiproliferative Role of TOB in T Cell Signaling | 0.012303 | Glutamate Metabolism | 6.61E-03 |
|  |  | Methionine Metabolism | 0.015136 | RAR Activation | 6.92E-03 |
|  |  | IGF-1 Signaling | 0.015849 | Type I Diabetes Mellitus Signaling | 6.92E-03 |
|  |  | NRF2-mediated Oxidative Stress Response | 0.016596 | Myc Mediated Apoptosis Signaling | 7.41E-03 |
|  |  | Production of Nitric Oxide and Reactive Oxygen Species in Macrophages | 0.016982 | Huntington's Disease Signaling | 7.76E-03 |
|  |  | Hypoxia Signaling in the Cardiovascular System | 0.018621 | Retinoic acid Mediated Apoptosis Signaling | 8.13E-03 |
|  |  | Insulin Receptor Signaling | 0.020893 | NF-κB Signaling | 8.91E-03 |
|  |  | Cell Cycle Regulation by BTG Family Proteins | 0.02138 | Nicotinate and Nicotinamide Metabolism | 9.55E-03 |
|  |  | GNRH Signaling | 0.02138 | Type II Diabetes Mellitus Signaling | 9.55E-03 |
|  |  | Sphingolipid Metabolism | 0.02138 | Erythropoietin Signaling | 9.77E-03 |
|  |  | DNA Methylation and Transcriptional Repression Signaling | 0.02138 | Melatonin Signaling | 9.77E-03 |
|  |  | Tight Junction Signaling | 0.021878 | Reelin Signaling in Neurons | 0.01 |
|  |  | TR/RXR Activation | 0.023442 | Assembly of RNA Polymerase III Complex | 0.010715 |
|  |  | LPS-stimulated MAPK Signaling | 0.023442 | Synthesis and Degradation of Ketone Bodies | 0.010715 |
|  |  | Rac Signaling | 0.024547 | Growth Hormone Signaling | 0.012303 |
|  |  | PTEN Signaling | 0.024547 | Production of Nitric Oxide and Reactive Oxygen Species in Macrophages | 0.01349 |
|  |  | EGF Signaling | 0.024547 | Methionine Metabolism | 0.013804 |
|  |  | TNFR2 Signaling | 0.026303 | Assembly of RNA Polymerase II Complex | 0.015488 |
|  |  | mTOR Signaling | 0.026915 | EIF2 Signaling | 0.015488 |
|  |  | Aldosterone Signaling in Epithelial Cells | 0.02884 | Protein Ubiquitination Pathway | 0.015849 |
|  |  | AMPK Signaling | 0.029512 | PI3K/AKT Signaling | 0.016218 |
|  |  | Glioma Signaling | 0.030903 | p38 MAPK Signaling | 0.020417 |
|  |  | ILK Signaling | 0.030903 | Rac Signaling | 0.020893 |
|  |  | Glucocorticoid Receptor Signaling | 0.030903 | PTEN Signaling | 0.020893 |
|  |  | EIF2 Signaling | 0.031623 | N-Glycan Biosynthesis | 0.02138 |
|  |  | OX40 Signaling Pathway | 0.031623 | IL-3 Signaling | 0.023988 |
|  |  | Cytotoxic T Lymphocyte-mediated Apoptosis of Target Cells | 0.034674 | Estrogen-Dependent Breast Cancer Signaling | 0.024547 |
|  |  | 14-3-3-mediated Signaling | 0.035481 | Xenobiotic Metabolism Signaling | 0.029512 |
|  |  | p70S6K Signaling | 0.036308 | Melanoma Signaling | 0.031623 |
|  |  | DNA Double-Strand Break Repair by Homologous Recombination | 0.037154 | Cardiac Hypertrophy Signaling | 0.033113 |
|  |  | IL-10 Signaling | 0.038019 | mTOR Signaling | 0.033884 |
|  |  | Glutamate Metabolism | 0.038019 | Melanocyte Development and Pigmentation Signaling | 0.033884 |
|  |  | PKCθ Signaling in T Lymphocytes | 0.039811 | Pyrimidine Metabolism | 0.034674 |
|  |  | Androgen Signaling | 0.040738 | DNA Double-Strand Break Repair by Homologous Recombination | 0.034674 |
|  |  | Role of PKR in Interferon Induction and Antiviral Response | 0.041687 | IGF-1 Signaling | 0.037154 |
|  |  | Cdc42 Signaling | 0.042658 | Angiopoietin Signaling | 0.038905 |
|  |  | Neurotrophin/TRK Signaling | 0.044668 | VEGF Signaling | 0.039811 |
|  |  | Lymphotoxin β Receptor Signaling | 0.045709 | Cell Cycle Control of Chromosomal Replication | 0.041687 |
|  |  | B Cell Receptor Signaling | 0.045709 | EGF Signaling | 0.043652 |
|  |  |  |  | Ceramide Signaling | 0.044668 |
|  |  |  |  | Docosahexaenoic Acid (DHA) Signaling | 0.045709 |
|  |  |  |  | CCR3 Signaling in Eosinophils | 0.046774 |
|  |  |  |  | Endometrial Cancer Signaling | 0.047863 |
