# Supplementary material for: IκBα mediates prostate cancer cell death induced by combinatorial targeting of the androgen receptor
Source: BMC Cancer. 2016 Feb 23;16:141. doi: 10.1186/s12885-016-2188-2 (PMC4785192; doi:10.1186/s12885-016-2188-2)
Supplement: Additional file 7: Table S4. — IPA network analysis for genes regulated by the combination when compared to individual doses of bicalutamide and vorinostat. (DOCX 18 kb) [file 12885_2016_2188_MOESM7_ESM.docx]

**Additional File 7 – Table S4: IPA network analysis for genes regulated by the combination when compared to individual doses of bicalutamide and vorinostat.**

Combination vs. Vorinostat

| **Score** | **Focus Molecules** | **Top Functions** | **Molecules in Network** |
| --- | --- | --- | --- |
| 46 | 24 | **Cell Morphology, Cellular Movement, Cell Signaling** | ABCG1,ADAMTS1,AKAP1,BAMBI,Collagen type I,Collagen type IV,Collagen(s),CYTH2,EHF,Fibrinogen,GNA15,HJURP,IL36A,KLK4,LAMA1,LAMA3,Laminin,LDL,MKI67,MMP1 (includes EG:300339),NFkB (complex),NPC1,Nr1h,PAK1IP1,Pdgf (complex),PDGF BB,PMAIP1,PMEPA1,RLN1/RLN2,SNAI2,Tgf beta,THBS1,TNFAIP8,TNFRSF10B,ZC3H12A |
| 40 | 22 | **Cellular Movement, Drug Metabolism, Endocrine System Development and Function** | Alpha catenin,ARHGAP1,ARHGAP6,Calpain,CAMKK2,ELL2,ERK1/2,FSH,GTPASE,hCG,HOMER2,IL6R,JAK,Lh,LIFR,MAF,NFKBIA,NRP1,PIAS1,Pias,PLC,PLCB1,PPAP2A,SGK1,SLC4A4,SOCS2,STAT,STAT5a/b,STEAP1,TBC1D8,TMEFF2,VCL,Vegf,ZBTB10,ZBTB16 |
| 36 | 20 | Cell Cycle, DNA Replication, Recombination, and Repair, Cellular Growth and Proliferation | 26s Proteasome,AMPK,Ap1,CLU,Cytochrome c,EFNA5,ERRFI1,Estrogen Receptor,F Actin,FASN,HMGCR,Hsp27,Hsp70,IGF1R,Insulin,KIF20A,KLK2,KLK3,KLK15,NKX3-1,p85 (pik3r),PI3K (complex),PLC gamma,POLK,Ras homolog,RHOU,SLC38A2,SLC6A8,SYNJ1,THRB,TPT1,Trypsin,TUBB,Ubiquitin,ZFHX3 |
| 27 | 16 | Lipid Metabolism, Small Molecule Biochemistry, Cancer | ABCC4,ACSL3,ANKRD11,C16orf53,C1orf21,CDC42EP3,CHD7,CYB5A,dihydrotestosterone,DPY30,EGFR ligand,FADS2,GFPT1,HPGD,HPGDS,KISS1R,LEP,MLL4,MT-CO3,MT-CYB,MT-ND4,Mup1 (includes others),NCOA1,NCOA4,PDE9A,PDLIM5,PMEPA1,PPARG,PTGER4,Relaxin,SDPR,SORD,TGFB1,TRPM8,UAP1 |
| 24 | 15 | Embryonic Development, Organismal Development, Cancer | ABHD2,alpha-estradiol,BAMBI,beta-estradiol,Ca2+,CTNNB1,CYP3A5,FAM105A,GREB1,GRHL2,GUCY1A3,GUCY2E,GUCY2F,HPGD,KCNN2,KLK5,LRRFIP2,MAK,mir-124,MYOF,NRP2,Pbsn,POLE2,PPM1K,PTP4A1,PTP4A2,REST,SEMA3C,SFRP2,SLC26A2,SMARCA4,STK17B,SYTL2,TCF7L1,WNT3 |
| 22 | 14 | Gene Expression, Infectious Disease, Lipid Metabolism | APPBP2,AR,CDKN2A,CENPN,CPS1,Cyp2c40 (includes others),DDC,DDIT4,EHF,EID3,EPHX1,FNBP1L,FOXA1,FOXD3,HUS1,HUS1B,hydrogen peroxide,KLK4,NAT1 (includes EG:116632),NR3C1,NSMAF,ONECUT1,ORC4,ORC5,Pbsn,PMEPA1,POU5F1,PPFIBP1,PTP4A2,Rad9-Rad1-Hus1,RAD9A,RAD9B,SCAP,SLC45A3,TP53INP1 |
| 20 | 13 | Cell Death, Cellular Development, Respiratory System Development and Function | ANKRA2,BDP1,BLOC1S1,CCP110,CENPO,EAF1,EWSR1,FAM167A,FXR2,HEATR6,HNF4A,KCNRG,KDM5C,KRR1,Mediator,MTMR9,NECAB2,PNMA1,POTEG/POTEM,PRUNE2,PTPN4,PTPN11,RB1,RNF40,SLC16A6,TADA1,TAF5L,TERT,TFAP2B,TFIIB,TIGD6,TNNT1,TRIP11,UBP1,UXT |
| 20 | 14 | Cellular Movement, Gene Expression, Cell Morphology | Akt,ASAP1,AVIL,C2orf3,DDIT3,DEGS1,ELK4,ERK,FGD4,Focal adhesion kinase,FYN,Gpcr,GSTM2,HBEGF,Histone h3,IgG,IKK (complex),IL1,Immunoglobulin,Interferon alpha,Jnk,Mapk,Mmp,Nfat (family),P38 MAPK,Pka,Pkc(s),Rac,Ras,SETMAR,SLC25A37,SNRPD1,SPSB1,SRC,TCR |
| 19 | 13 | Genetic Disorder, Respiratory Disease, Cellular Assembly and Organization | Actin,APP,ARHGEF26,ATP2B2,CACNA2D1,Caspase,DLG4,DNAH1,DNAH5,DNAH9,DNAH10,DNAH14,DNAI1,DNALI1,FAM65B,heparin,HOMER2,HTT,ICAM1,IL5,LIPH,NSF,OSTF1,PHF8,PRDX4,PTPN5,RAB6C/WTH3DI,RASSF3,RASSF4,SAV1,SGK223,SLC7A11,SNX24,STK4,WAC |
| 18 | 12 | Molecular Transport, Nucleic Acid Metabolism, Small Molecule Biochemistry | C11orf82,CHAC1,CMIP,CRYAA,DDR2,DNAJA3,DNAJB14,DNAJC3,DNAJC7,DTX3L,GABA,GBP1,GRASP,HSP,HSPA1L,IFIT5,IFNG,IREB2,LRCH1,MICAL1,PARP9,PCTP,PDE4A,PHACTR2,PRDX2,SLC36A1,SRC,TRIM8,TRIM25,TRPC4,UBE2D1,UBE2R2,VLDLR,ZFP36,ZFX |
| 14 | 10 | DNA Replication, Recombination, and Repair, Gene Expression, Cell Signaling | ALDH9A1,Calmodulin,CCNH,Ck2,EIF2S2,ELK3,HEBP2,JAG1,KCNN1,KCNN2,KCNN3,LIMCH1,MFAP2,miR-124,miR-21/miR-590-5p,MTAP,PHLPP1,PHLPP2,PODXL,PPFIBP1,RAD51AP1,RNA polymerase II,RPRM,SNAI2,SPHK2,THBS2,TOP1,TP53,TRIM13,TRIM29,Troponin t,TRPV4,Tubulin,USP39,USP46 |
| 2 | 1 | Cell Death, Gene Expression, Cellular Development | LOC100287275/PCOTH,TAF1B |
| 2 | 1 | Behavior, Skeletal and Muscular System Development and Function, Cell Signaling | C1orf116,HOMER1 |
| 2 | 1 | Cell Cycle, Cellular Assembly and Organization, DNA Replication, Recombination, and Repair | CEP120,SPICE1 |
| 2 | 1 | Genetic Disorder, Neurological Disease, Organismal Injury and Abnormalities | ADAM7,ITM2B |
| 2 | 1 | Neurological Disease, Psychological Disorders, Cell-To-Cell Signaling and Interaction | RBFOX1,RBM24 |
| 2 | 1 | Cellular Assembly and Organization, Nervous System Development and Function, Psychological Disorders | LRRC4C,NTNG1 |
| 2 | 1 | Cellular Assembly and Organization, Cellular Compromise, DNA Replication, Recombination, and Repair | FBXO38,KLF7,USP7 |

Combination vs. Bicalutamide

| **Score** | **Focus Molecules** | **Top Functions** | **Molecules in Network** |
| --- | --- | --- | --- |
| 46 | 24 | **Cell Morphology, Cellular Movement, Cell Signaling** | ABCG1,ADAMTS1,AKAP1,BAMBI,Collagen type I,Collagen type IV,Collagen(s),CYTH2,EHF,Fibrinogen,GNA15,HJURP,IL36A,KLK4,LAMA1,LAMA3,Laminin,LDL,MKI67,MMP1 (includes EG:300339),NFkB (complex),NPC1,Nr1h,PAK1IP1,Pdgf (complex),PDGF BB,PMAIP1,PMEPA1,RLN1/RLN2,SNAI2,Tgf beta,THBS1,TNFAIP8,TNFRSF10B,ZC3H12A |
| 40 | 22 | **Cellular Movement, Drug Metabolism, Endocrine System Development and Function** | Alpha catenin,ARHGAP1,ARHGAP6,Calpain,CAMKK2,ELL2,ERK1/2,FSH,GTPASE,hCG,HOMER2,IL6R,JAK,Lh,LIFR,MAF,NFKBIA,NRP1,PIAS1,Pias,PLC,PLCB1,PPAP2A,SGK1,SLC4A4,SOCS2,STAT,STAT5a/b,STEAP1,TBC1D8,TMEFF2,VCL,Vegf,ZBTB10,ZBTB16 |
| 36 | 20 | Cell Cycle, DNA Replication, Recombination, and Repair, Cellular Growth and Proliferation | 26s Proteasome,AMPK,Ap1,CLU,Cytochrome c,EFNA5,ERRFI1,Estrogen Receptor,F Actin,FASN,HMGCR,Hsp27,Hsp70,IGF1R,Insulin,KIF20A,KLK2,KLK3,KLK15,NKX3-1,p85 (pik3r),PI3K (complex),PLC gamma,POLK,Ras homolog,RHOU,SLC38A2,SLC6A8,SYNJ1,THRB,TPT1,Trypsin,TUBB,Ubiquitin,ZFHX3 |
| 29 | 17 | Energy Production, Lipid Metabolism, Small Molecule Biochemistry | ABCC4,ACSL3,ANKRD11,C16orf53,C1orf21,CCR10,CDC42EP3,CHD7,CYB5A,dihydrotestosterone,DPY30,EGFR ligand,EHHADH,HPGD,HPGDS,KISS1R,LEP,LIFR,MLL4,MT-CO3,MT-CYB,Mup1 (includes others),MYF6,NCOA1,NCOA4,PDE9A,PDLIM5,PMEPA1,PPARG,PTGER4,Relaxin,SORD,TGFB1,TRPM8,UAP1 |
| 24 | 15 | Nucleic Acid Metabolism, Small Molecule Biochemistry, Embryonic Development | ABHD2,BAMBI,beta-estradiol,Ca2+,Calbindin,CTNNB1,CYP3A5,FAM105A,GC,GREB1,GRHL2,GUCY1A3,GUCY2D,GUCY2E,GUCY2F,HPGD,KCNN2,LRRFIP2,MAK,mir-124,MYOF,NRP2,Pbsn,POLE2,PPM1K,REST,SEMA3C,sGC,SLC26A2,SMARCA4,SPN,STK17B,SYTL2,TCF7L1,WNT3 |
| 22 | 14 | Gene Expression, Infectious Disease, Lipid Metabolism | ALDOB,APPBP2,AR,CDKN2A,CENPN,CPS1,Cyp2c40 (includes others),DDC,EHF,EID3,EPHX1,FNBP1L,FOXA1,FOXD3,HUS1,HUS1B,hydrogen peroxide,KLK4,LIG1,NAT1 (includes EG:116632),NR3C1,NSMAF,ONECUT1,ORC5,Pbsn,PMEPA1,POU5F1,PPFIBP1,PTP4A2,RAD1,Rad9-Rad1-Hus1,RAD9A,RAD9B,SLC45A3,TP53INP1 |
| 20 | 13 | Gene Expression, Cell Death, Cellular Development | ANKRA2,BDP1,BLOC1S1,CCP110,CENPO,CNN1,EWSR1,FAM167A,FLOT1,FXR2,HEATR6,HNF4A,KCNRG,KDM5C,KRR1,Mediator,MTMR9,NECAB2,PNMA1,POTEG/POTEM,PRUNE2,PTPN4,PTPN11,RB1,RNF40,SLC16A6,TADA1,TAF5L,TERT,TFAP2B,TFIIB,TIGD6,TNNT1,TRIP11,UBP1 |
| 20 | 14 | Cellular Movement, Gene Expression, Cell Morphology | Akt,ASAP1,AVIL,C2orf3,DDIT3,DEGS1,ELK4,ERK,FGD4,Focal adhesion kinase,FYN,Gpcr,GSTM2,HBEGF,Histone h3,IgG,IKK (complex),IL1,Immunoglobulin,Interferon alpha,Jnk,Mapk,Mmp,Nfat (family),P38 MAPK,Pka,Pkc(s),Rac,Ras,SETMAR,SLC25A37,SNRPD1,SPSB1,SRC,TCR |
| 18 | 12 | Gene Expression, Amino Acid Metabolism, Molecular Transport | ABL2,AGRN,C11orf82,CHAC1,CMIP,CRYAA,DDR2,DNAJB14,DNAJC3,DNAJC7,DTX1,DTX3L,EVL,GABA,HSP,IFIT5,IFNG,IREB2,LRCH1,MICAL1,PARP9,PCTP,PDE4A,PDE4D,PHACTR2,SLC36A1,SRC,TRIM8,TRIM25,TRPC4,UBE2D1,UBE2E2,UBE2R2,ZFP36,ZFX |
| 17 | 12 | Gastrointestinal Disease, Genetic Disorder, Respiratory Disease | Actin,APP,ARC,ARHGEF26,CACNA2D1,Caspase,DLG4,DNAH1,DNAH5,DNAH6,DNAH8,DNAH9,DNAH10,DNAH14,DNAI1,DNALI1,FAM65B,heparin,HTT,ICAM1,IL5,LIPH,NSF,OSTF1,PHF8,PRDX4,PTPN5,RAB6C/WTH3DI,RASSF3,SGK223,SLC7A11,SNX24,STK4,THY1,WAC |
| 14 | 10 | DNA Replication, Recombination, and Repair, Gene Expression, Cellular Movement | ALDH9A1,Calmodulin,Ck2,ELK3,HEBP2,JAG1,KCNN1,KCNN2,KCNN3,LIMCH1,MAP2,MED14,MFAP2,miR-124,miR-21/miR-590-5p,MTAP,PHLPP2,PODXL,PPFIBP1,PTPRA,RAD51AP1,RNA polymerase II,RPRM,S100B,SNAI2,SPHK2,THBS2,TOM1L1,TP53,TRIM13,Troponin t,TRPV4,Tubulin,USP46,XRCC1 |
| 2 | 1 | Cell Death, Gene Expression, Cellular Development | LOC100287275/PCOTH,TAF1B |
| 2 | 1 | Behavior, Skeletal and Muscular System Development and Function, Cell Signaling | C1orf116,HOMER1 |
| 2 | 1 | Cell Cycle, Cellular Assembly and Organization, DNA Replication, Recombination, and Repair | CEP120,SPICE1 |
| 2 | 1 | Genetic Disorder, Neurological Disease, Organismal Injury and Abnormalities | ADAM7,ITM2B |
| 2 | 1 | Neurological Disease, Psychological Disorders, Cell-To-Cell Signaling and Interaction | RBFOX1,RBM24 |
| 2 | 1 | Cellular Assembly and Organization, Nervous System Development and Function, Psychological Disorders | LRRC4C,NTNG1 |
| 2 | 1 | Cellular Assembly and Organization, Cellular Compromise, DNA Replication, Recombination, and Repair | FBXO38,KLF7,USP7 |
